# Differential A-to-I editing of SINE B2 RNAs unveils an epitranscriptome response to Aβ neurotoxicity

**DOI:** 10.26508/lsa.202603668

**Published:** 2026-06-16

**Authors:** Liam Mitchell, Luke Saville, Babita Gollen, Travis Haight, Riya Roy, Cody Turner, Yubo Cheng, Igor Kovalchuk, Majid H Mohajerani, Athanasios Zovoilis

**Affiliations:** 1 Department of Biochemistry and Medical Genetics, University of Manitoba , Winnipeg, Canada; 2 Paul Albrechtsen Research Institute CCMB, Winnipeg, Canada; 3 Department of Neuroscience, Canadian Centre for Behavioral Neuroscience, University of Lethbridge , Lethbridge, Canada; 4 Department of Biological Sciences, University of Lethbridge , Lethbridge, Canada; 5 Department of Psychiatry, McGill University, Montreal, Canada; 6 Douglas Hospital Research Center, Montreal, Canada

## Abstract

Adenosine-to-inosine (A-to-I) RNA editing is a major epitranscriptomic mechanism, yet its contribution to non-coding RNA regulation during neurodegeneration is largely unknown. SINE B2 RNAs represent the dominant editing substrates in mice and have been shown to regulate gene expression. Here, we introduce and validate a repeat-aware bioinformatics framework that enables position-specific quantification of A-to-I editing within SINE RNAs, which has been challenging using standard genome-based pipelines. Applying this approach, we identify discrete editing hotspots in mouse SINE B2 RNAs that are selectively increased during early amyloid beta pathology in independent mouse models and in hippocampal neurons exposed to amyloid beta toxicity. Functional perturbation of ADAR activity alters both B2 RNA editing levels and the expression of B2 RNA–regulated genes, directly linking RNA editing to SINE-mediated transcriptional control. Nanopore sequencing confirmed increased RNA modification signals at these regions. Together, our findings establish a previously unrecognized epitranscriptomic response to amyloid beta neurotoxicity mediated by site-specific A-to-I editing of SINE RNAs.

## Abstract (precis)

The study identifies site-specific SINE B2 RNA editing as an early epitranscriptomic response to amyloid beta neurotoxicity in the mouse hippocampus.

## Introduction

Alzheimer’s disease (AD) constitutes one of the most frequent neurodegenerative and aging-associated diseases. A large number of studies have elucidated various cell pathology aspects of AD, with amyloid beta neuro pathology being one of the required features for the diagnosis of AD, and amyloid beta peptides being associated with AD pathogenesis in both human and rodent models (Ittner et al, 2010; Bloom, 2014; Gonzalez et al, 2018; Mehla et al, 2019). However, many of the molecular processes underlying the cellular response to amyloid beta in neural cells remain elusive, which potentially hampers efforts for better molecular diagnostics and therapeutics for this debilitating disease (Bertram & Tanzi, 2008; Korczyn, 2012; Toledo et al, 2013; Hane et al, 2017). Among molecular processes that have attracted limited attention to date are potential epitranscriptome changes associated with amyloid beta pathology.

Currently, across the various domains of life, at least 163 different RNA modifications have been described, constituting a significant part of what is known as the epitranscriptome (Boccaletto et al, 2018). However, many of their functions are not well understood (Li & Mason, 2014). A common type of RNA modification in mammalian epitranscriptomes is RNA editing involving adenosine-to-inosine conversions (A-to-I editing) (Levanon et al, 2004; Neeman et al, 2006; Khermesh et al, 2016; Wang et al, 2017; Nakahama & Kawahara, 2020). A-to-I editing is catalyzed by the ADAR (adenosine deaminases acting on RNA) family of enzymes, which act upon dsRNA structures (Wagner et al, 1989; Stefl et al, 2006; Nishikura, 2016) and catalyze the conversion of adenosine to inosine by hydrolytic deamination (Bass & Weintraub, 1988; Wagner et al, 1989), with ADAR1, encoded in mouse by the *Adar* gene, and ADAR2, encoded by the *Adarb1* gene, being the ones responsible for the vast majority of A-to-I editing (ADAR3 encoded by *Adarb2* lacks such activity) (Lehmann & Bass, 2000; Hundley & Bass, 2010; Kapoor et al, 2020). These A-to-I edits have been shown to influence directly and indirectly the stability of RNA (Morita et al, 2013; Nishikura, 2016; Solomon et al, 2017) and act as signals that distinguish endogenous RNAs from viral ones and protect them from cellular defense mechanisms and destabilization (Samuel, 2012; Liddicoat et al, 2015; Tomaselli et al, 2015). In addition, deficiencies in A-to-I RNA editing have been associated with diseases, including neurodegenerative diseases (Nishikura, 2016). In particular, RNA editing in protein-coding genes has been shown to alter codon usage of genes associated with synaptic function (Singh, 2012), Alzheimer’s disease (Khermesh et al, 2016; Annese et al, 2018), prion diseases (Kanata et al, 2019) and other neurodegenerative diseases (Krestel & Meier, 2018; Larsen et al, 2018). However, beyond recoding, little is known about the role of A-to-I editing of non-coding RNAs and particularly SINE RNAs in amyloid pathology, despite SINE RNAs constituting the primary constituents of A-to-I editing in mammalian transcriptomes (Kim et al, 2004; Neeman et al, 2006; Paz-Yaacov et al, 2010; Porath et al, 2017; Larsen et al, 2018).

SINE RNAs are non-coding RNAs generated by repetitive genomic elements called short interspersed nuclear elements (SINEs). SINEs include B2 elements in mice and Alu elements in humans, which are among the most frequent repeats in the respective genomes and are transcribed either independently by RNA polymerase III or as part of RNA polymerase II transcripts in which they are embedded. SINE RNAs constitute one of the primary targets of A-to-I editing (Levanon et al, 2004; Neeman et al, 2006; Chen & Carmichael, 2009; Porath et al, 2017; Huang et al, 2018), which is a process that has been described as essential in marking the RNA as endogenous, and thus, not eliciting an endogenous interferon response as viral RNAs will (Wang et al, 2017). Despite being considered initially as genomic parasites due to their transposon origin (Karijolich et al, 2017), multiple recent studies have revealed important functions for SINE genomic elements ranging from the generation of new regulatory genomic elements to cryptic splicing sites and RNA circularization (Lin et al, 2008; Jeck et al, 2013; Kaaij et al, 2019; Zhang et al, 2019). The same applies to SINE RNAs produced by these genomic elements, which have initially been considered transcriptional noise generated by the activation of these transposons during cellular stress. However, earlier work from the Goodrich and Kugel labs demonstrated the ability of SINE RNAs to bind and suppress RNA polymerase II, and thus, marked SINE RNAs as key regulators of gene expression (Espinoza et al, 2007; Mariner et al, 2008; Yakovchuk et al, 2009; Ponicsan et al, 2010, 2015). This activity mediates the transcriptome-wide suppression of housekeeping genes in response to stress, thereby facilitating the redistribution of resources towards pro-survival pathways (Mariner et al, 2008).

Subsequently, we revealed that SINE RNAs regulate the activation of gene expression during the cellular response to stress. By occupying stress-response genes (B2 SRGs) and suppressing RNA polymerase II in the pre-stimulus state, B2 RNAs keep these genes poised for fast activation during stress (Zovoilis et al, 2016). Upon application of a stress stimulus, B2 RNAs, which are self-cleaving RNAs (Hernandez et al, 2020), interact with proteins such as EZH2 and HSF1 (Zovoilis et al, 2016; Cheng et al, 2020), which accelerate their processing, leading to the release of RNA polymerase II suppression and the activation of pro-survival genes. As a result, SINE RNAs in mice act as transcriptional switches during the response to stress stimuli by binding RNA polymerase II at several stress-response genes in the pro-cellular stress state and suppressing their transcription.

Interestingly, in a previous study, we found that the above B2 RNA–dependent mode of gene regulation is impaired in hippocampal tissues during amyloid beta pathology. In particular, amyloid toxicity leads to increased destabilization of B2 RNAs in the absence of a stimulus, and thus, to persistent hyperactivation of B2-regulated stress-response genes (B2 SRGs), which, in turn, are associated with activation of pro-apoptotic pathways (Cheng et al, 2020). Given the role of A-to-I editing in regulating RNA stability and that A-to-I editing has been associated with SINE RNAs, we decided to investigate how exactly A-to-I editing is distributed across B2 RNAs, how it is modified in response to amyloid beta pathology, and whether it is associated with transcriptome changes in B2 SRGs. However, investigating the potential role of B2 RNA editing in this biological context first requires an analytic framework capable of accurately capturing A-to-I events within these transcripts. The repetitive nature of B2 RNAs and the substantial sequence heterogeneity among individual SINE B2 copies pose major challenges for standard RNA editing analysis pipelines, which were primarily developed for uniquely mappable genomic regions. These limitations hinder reliable quantification of editing at specific base positions of B2 RNAs. Therefore, a second key objective of the present study is to establish a computational strategy tailored to repetitive SINE RNAs that enables robust detection of position-specific A-to-I editing events in B2 RNAs, before further examination of these RNAs in the context of pathology.

## Results

### A method for estimating relative A-to-I editing rate across SINE B2 RNAs

For the estimation of A-to-I editing, we used Illumina-based next-generation sequencing as the most widely used approach. Using RNA sequencing (RNA-seq), we can interpret A-to-I editing as an A-to-G substitution that occurs during cDNA synthesis during library construction. Data analysis typically involves mapping sequenced reads against the genome and identifying A-to-G sequence conversions relative to the reference sequence that could correspond to potential A-to-I events. The editing rate is then calculated at each genomic position for each transcript. This approach, using REDI tools among other tools (Picardi & Pesole, 2013), has been previously applied to map the exact editing locations in various protein-coding genes and non-repetitive non-coding sequences, and in the case of RNAs transcribed from repetitive elements, to estimate a “bulk” editing rate for entire repeat families (Picardi et al, 2017). However, in the case of RNAs from repetitive elements, such as SINE B2 RNAs, due to their high sequence variability and repetitive nature, such an estimation of their A-to-I editing rate is complicated by a number of parameters. Firstly, SINE B2 RNA sequences are derived from thousands of different loci. The majority of them share identical sequences with other B2 elements located at multiple distinct loci. Thus, mapping of a sequenced read against the genome would often assign a read to multiple locations. To address this problem, many current approaches either filter out reads that are not uniquely mapped to the genome or randomly select only one of the multiple genomic locations that best map the read. However, this approach results in either discarding a large number of potential SINE-mapping reads or misassigning them to the wrong genomic position. The latter could result in SNPs at a specific SINE locus being interpreted falsely as an editing event because B2 elements, despite sharing high sequence similarity with the consensus sequence, also harbor multiple SNPs, small insertions, or deletions relative to this sequence.

**Figure 1.**
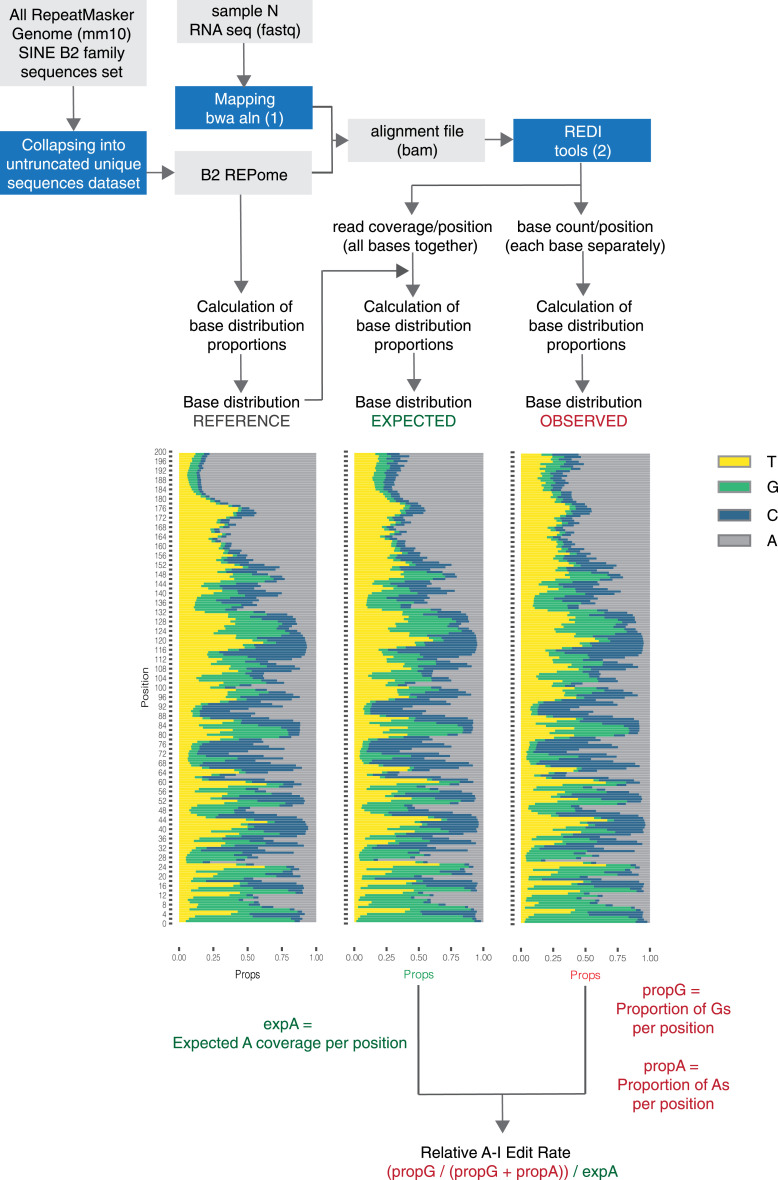
Workflow describing the method for estimating relative A-to-I edit rate across SINE B2 RNAs. Arrows depict the flow of analysis. Plots depict the distribution of base proportions (for A, T, C, G) across B2 RNAs based on (i) sequences in the reference (Repeat Masker, mm10) (REFERENCE—left plot), (ii) read coverage in each sample with regard to the reference (EXPECTED—middle plot) and (iii) actual base coverage observed in each sample (OBSERVED—right plot). The Y-axis of each plot represents the position in a B2 metagene combining all unique B2 RNA sequences (REPome) aligned at the start site of their consensus sequence with numbers representing the distance from the sequence start (+1). The way the editing rate is calculated is shown in the displayed formula, combining expected and observed base proportions per position and sample. Further details on the calculations of base distributions and of the relative edit rate are provided in Methods. Props: cumulative proportions of four bases (cumulative proportion of all bases added together = 1). (1) as described in Li et al (2009), (2) as described in Picardi and Pesole (2013).

To circumvent the above problems, we modified the above standard A-to-I analysis approach by adjusting the mapping step and introducing an additional analysis step. An overview of this approach is presented in Fig 1 and described in detail in the methods section. In brief, to account for the obstacles that multiple genomic mapping poses, instead of mapping reads against the mouse reference genome, we have mapped them against a unique “REPome” of SINE B2 sequences. This includes all B2 repetitive elements of a certain family identified by Repeat Masker for assembly version mm10, with the same sequences mapping to multiple loci collapsed into one unique sequence each time. This results in only one sequence with a unique ID regardless of the number of its instances in the genome. In essence, we treat all RNAs generated by multiple different sets of genomic elements sharing the same sequence as a single pool of RNAs studied together. Subsequently, to account for diversity among the different members of the B2 REPome with respect to the B2 consensus sequence, we have implemented an RNA-seq population-based approach. In this approach, instead of calculating the RNA editing for every RNA molecule that is a member of the B2 REPome separately, we calculate proportions of base compositions across the entire population of B2 RNAs. This results in the construction of a reference distribution and two sample-specific distributions of base compositions across the length of a B2 RNA metagene model representing all B2 RNAs in the REPome, aligned with regard to their start position. The reference distribution is the distribution calculated by the reference sequences present in the REPome, independent of the sequenced sample (Fig 1, left plot, annotated as REFERENCE). The second one is the expected distribution if no A-to-I editing occurs, calculated sample-wise from a subset of the first distribution based on the read coverage at each position for each sample (Fig 1, middle plot, annotated as EXPECTED). This distribution is sample-dependent. The third distribution is the actual distribution observed in the reads from each sample (Fig 1, right plot, annotated as OBSERVED) and is also sample-dependent. Based on the last distribution, a ratio of guanosines to the sum of adenosines and guanosines, which represent potential A-to-I events, is calculated for every position and sample. This rate is weighted by the number of expected adenosines at that position to narrow the identification of A-to-I events in positions with frequently occurring adenosines (Fig 1).

### *Adar/Adarb1* knockdown confirms the ability of our customized A-to-I editing analysis to effectively identify A-to-I editing across B2 RNAs

**Figure 2.**
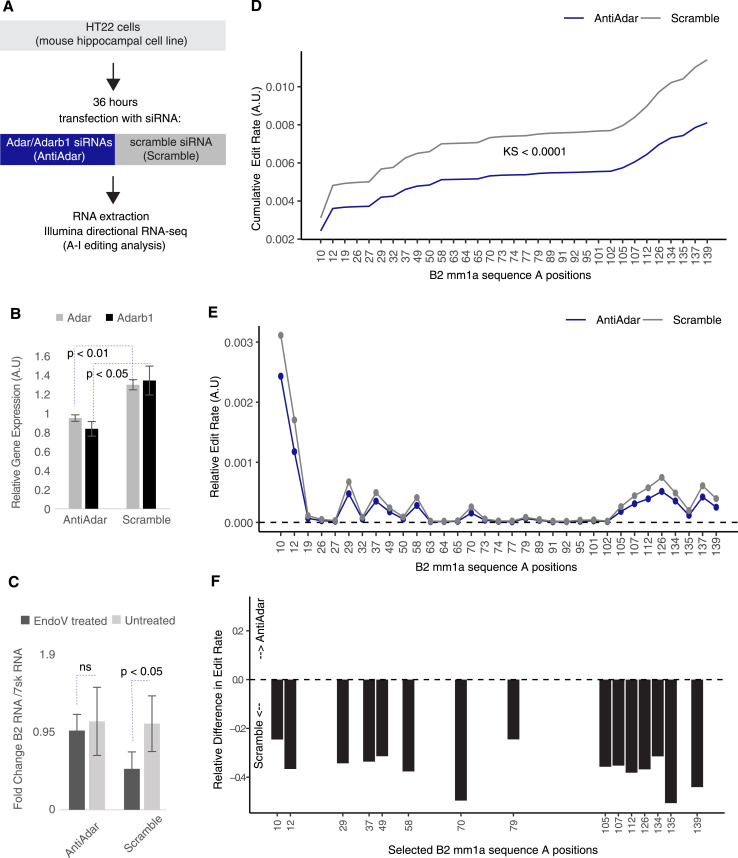
Knockdown of *Adar* and *Adarb1* reduces A-to-I editing of B2 RNAs in hippocampal cells. **(A)** Experimental design for siRNA-mediated knockdown of *Adar* and *Adarb1* (AntiAdar) or a scramble control in HT-22 cells (Scramble). Four biological replicates were used per group. **(B)** Successful *Adar and Adarb1* KD under the transfection conditions used in this study. RT-qPCR quantification of *Adar and Adarb1* mRNA levels (normalized to *Hprt* expression) in an independent set of experiments. Error bars denote SD (n = 3, one-tailed *t*-test). **(C)** Endonuclease V assay showing fold-change differences in B2 RNA levels in Scramble but not AntiAdar samples, measured using RT-qPCR and normalized to *7SK*. Error bars denote SD (n = 4 per group, one-tailed *t*-test, ns:non-significant). **(D)** Cumulative edit rate distributions across B2 RNAs (mm1a) in AntiAdar and Scramble groups (mean per group per position, Kolmogorov–Smirnov test value shown). The X-axis shows one value per adenosine position in the B2 mm1a consensus sequence (AU, arbitrary units). **(E)** Position-specific mean relative edit rate across all consensus A positions in the B2 mm1a sequence (AU, arbitrary units). **(F)** Relative differences in edit rate (AntiAdar–Scramble) at positions with relative edit rate > 0.0001 (see panel (C)); negative values denote higher editing in Scramble controls. X-axis has one value per adenosine position in the B2 mm1a consensus sequence.

To assess the performance of our customized pipeline in detecting A-to-I editing in SINE B2 RNAs, we performed, in the hippocampal cell line HT-22, siRNA-mediated knockdown of the transcripts of the two main A-to-I editing enzymes, *Adar* for ADAR1 *and Adarb1* for ADAR2, with their combined knockdown referred as “AntiAdar” throughout the text. The above cell line has previously been used by us and others as a model of amyloid beta-induced toxicity in hippocampal-derived neurons (Zhang et al, 2018; Cheng et al, 2020). Cells treated with a scramble siRNA (Scramble) served as a control. The experimental design is outlined in Fig 2A.

**Figure S1.**
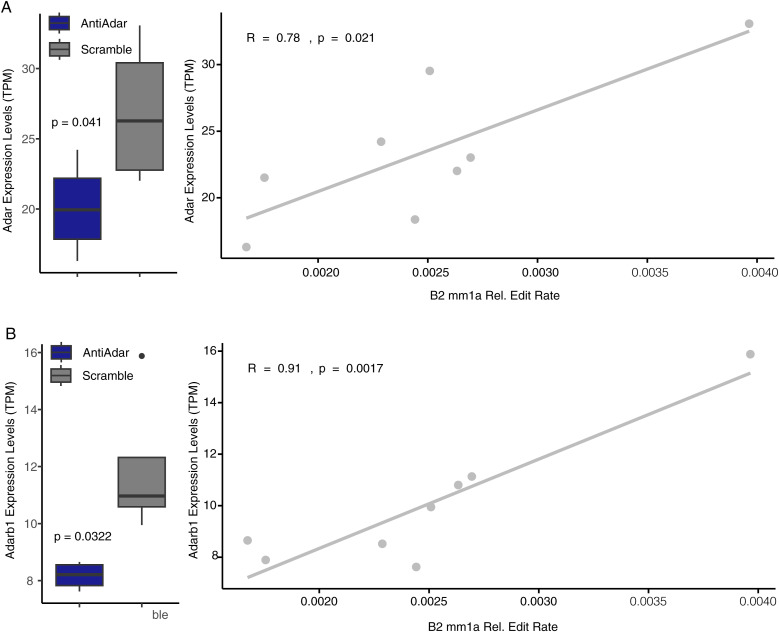
Sample-wise correlations between Adar/Adarb1 expression and A-to-I edit rates in siRNA-treated HT-22 cells (see Fig 2). **(A, B)** Left panels show transcript per million expression of *Adar* (A) and *Adarb1* (B) as measured through RNA-seq. *P*-values in (A) are derived from a one-tailed *t*-test. Right panels display as per replicate Pearson correlations between *Adar* or *Adarb1* transcript abundance and cumulative edit rate across the subset of highly edited (selected) A positions (Panel (A): R = 0.78, *P* = 0.021; Panel (B): R = 0.91, *P* = 0.0017).

Knockdown efficiency was validated by RT-qPCR, confirming down-regulation of both *Adar* and *Adarb1* transcripts (Fig 2B). To further substantiate these results, we performed an assay using Endonuclease V (EndoV), which specifically cleaves inosine-containing RNAs (Morita et al, 2013). The processed products were then assessed by RT-qPCR against B2 RNAs. In Scramble controls, EndoV treatment reduced full-length B2 RNA levels (normalized to 7SK), whereas this reduction was abolished in AntiAdar-treated cells, consistent with the depletion of inosine-bearing transcripts (Fig 2C). Overall, the successful knockdown of *Adar* and *Adarb1* transcripts, which was also confirmed through RNA-seq (Fig S1A and B), was highly correlated with changes in relative edit rates of B2 RNA transcripts (Fig S1A and B).

Then, using our customized pipeline, we calculated the relative A-to-I edit rate at B2 RNA positions for each sample and the average rate within each group (Scramble, AntiAdar). In this study, we focused specifically on members of the mm1a B2 RNA subfamily, as mm1a—and the closely related to it mm1t subfamily—represent the subfamilies that most faithfully correspond to the canonical B2 RNA consensus previously used in the literature to study B2 RNAs suppressive effect on RNA polymerase II (Espinoza et al, 2007). Subsequently, we visualized the cumulative distributions of A-to-I editing across all those B2 RNA positions containing an adenosine—the potential substrate for ADAR1/2 activity—and confirmed that AntiAdar treated samples displayed significantly lower relative edit rates compared with scramble siRNA controls (Scramble) (Fig 2D). Complementing this cumulative view, Fig 2E depicts relative edit rates at each individual adenosine position, highlighting discrete peaks corresponding to specific editing sites. Positions of these peaks (corresponding to a relative edit rate >10^−4^) are shown in Fig 2F. Other positions outside these hotspots did not exhibit significant relative edit rates (<10^−4^) and were not analyzed further. The amplitude of the peaks at the identified hotspots was markedly reduced after AntiAdar treatment, depicting a relative difference between AntiAdar and Scramble between −0.2 and −0.5, confirming that the identified A-to-I conversions at these positions depend on ADAR1/2 activity (Fig 2F).

The positional distribution of editing events identified by our analysis reveals non-uniformity of A-to-I editing along the B2 RNA as editing was concentrated at specific hotspots that are often clustered together. Interestingly, several of the observed peaks/peak clusters coincide with B2 RNA regions predicted in previous studies to form double-stranded RNA structures (Espinoza et al, 2007; Podbevsek et al, 2018; Sharma et al, 2024), particularly within the first ∼75 nucleotides and in double-strand domains near the 3′ end.

**Figure S2.**
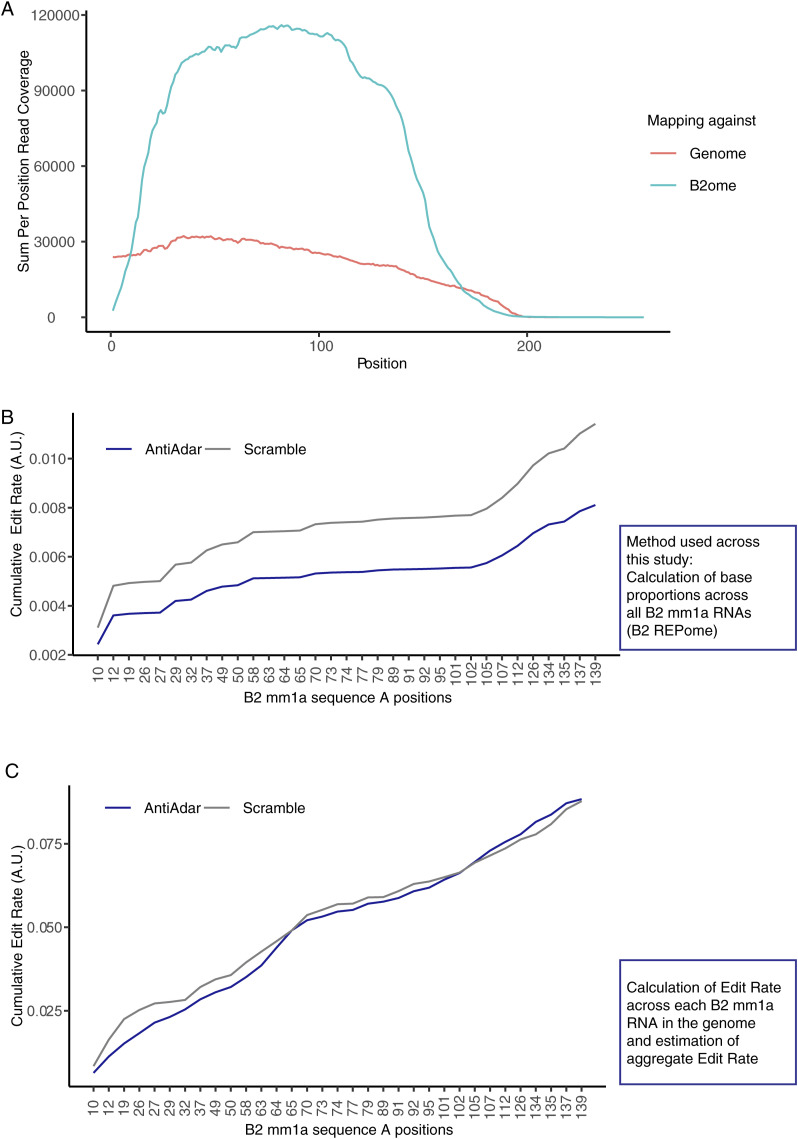
Our customized approach for calculating A-to-I edit rates achieves higher fidelity than standard genome-aligned methods. Two methodological distinctions underlie this improvement. In typical Illumina-based A-to-I editing detection, reads are aligned to the genome and intersected with annotated elements of interest “alignment on genome.” **(A)** In contrast, our method aligns reads directly to the unique transcriptome of the elements of interest “alignment on unique B2”; panel (A). A second distinction lies in the stage at which values are aggregated. The standard approach calculates editing rate per reference transcript and subsequently aggregates these across transcripts to derive a sample-level value “edit-then-group.” Our method first aggregates the transcripts and then computes the editing rate across them collectively “group-then-edit-rate.” Comparisons were performed using the same data as Fig 2 (n = 4 per group). Alignment on genome was performed using the same alignment parameters as for unique B2, see the Materials and Methods section. **(A)** Differences in the alignment strategy. Genome-based alignment yields reduced read coverage across repetitive B2 elements, whereas alignment to the unique B2-ome achieves markedly higher base coverage. **(B)** The customized “group-then-edit-rate” approach, as applied throughout the study (identical to Fig 2B – presented here to facilitate comparison with panel (C)), reveals reduced editing in AntiAdar compared with Scramble samples, consistent with qPCR results for *Adar/Adarb1* mRNA levels (Fig 2E and F and nanopore data–Fig 4). **(C)** The same dataset was analyzed using the typical approach. Editing rate is computed using the conventional A-to-I formula *G/(A+G)* across reference positions containing an A. The overall trend diverges from *Adar/Adarb1* expression patterns, showing minimal difference between conditions and even a mild apparent increase in AntiAdar samples, which are expected to exhibit lower editing. There is also no positional divergence in editing rates across the different positions.

Finally, we compared the efficiency of our repeat-based, customized pipeline for identifying A-to-I editing across B2 RNAs with that of the standard genome-based approach. As shown in Fig S2A, standard genome-based alignment (used as a first step for capturing editing events) failed to capture many reads that our B2 REPome (B2-ome)-based alignment method was able to capture. Fig S2B illustrates how our customized approach accurately resolves position-specific differences in editing between treated and control cells compared with that of the standard genome-based method (Fig S2C), which produces a potentially artificial, homogeneous editing pattern (depicted as an almost straight line in the cumulative distribution), lacking the distinct positional differences that would be expected from ADAR-dependent editing activity. It also fails to accurately quantify the editing differences between treated and untreated samples. This loss of positional specificity and overall sensitivity stems from the standard method’s inability to distinguish true editing from sequence variation among the numerous B2 repeat copies. These results underscore the improved accuracy of our customized method for quantifying A-to-I editing in repetitive elements such as B2 RNAs.

### B2 RNA editing is increased during the first stages of amyloid beta pathology in the mouse hippocampus

**Figure 3.**
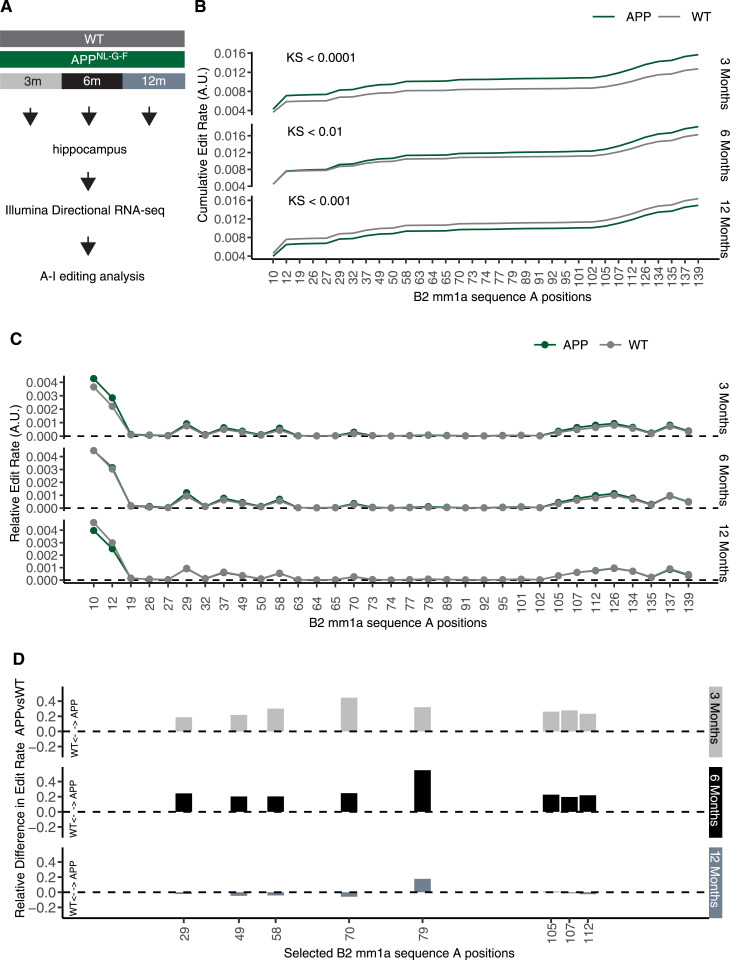
A-to-I editing increases at specific B2 RNA positions during early amyloid beta pathology. **(A)** Experimental overview of hippocampal RNA-seq datasets from APP^NL-G-F^ transgenic (APP) and WT mice at 3, 6, and 12 mo of age from reference Cheng et al (2020). m denotes the age of mice in months (n = 3 per treatment at each age group, except for 3-mo WT mice, which were n = 2). **(B)** Cumulative distributions of editi rates across B2 RNAs (mm1a) at each time point (Kolmogorov–Smirnov test values shown) (AU, arbitrary units). **(C)** Position-specific mean relative edit rates in APP and WT hippocampi at each age (AU, arbitrary units). **(D)** Relative differences (APP–WT) in edit rates at consensus A positions of Fig 2D that differ significantly between APP and WT (Fig S3). Positive values indicate higher editing in APP.

Using our customized SINE A-to-I editing analysis, we then investigated whether and how B2 RNA editing levels change in response to amyloid beta pathology. For this purpose, we used the same transcriptome dataset used in our previous study for establishing the relationship between B2 RNA processing, target gene hyperactivation, and amyloid beta pathology in the mouse hippocampus (Cheng et al, 2020). This dataset comprises directional Illumina RNA-seq data from the hippocampus of a transgenic mouse model of amyloid beta pathology APP^NL-G-F^ (APP) (Saito et al, 2014), compared with the corresponding WT control (C57BL/6J). This mouse model has been extensively described by us and others with regard to the progression of learning impairment over time and tested for amyloid beta load, RNA expression profiles, and B2 RNA processing ratio (Mehla et al, 2019; Cheng et al, 2020). The studied APP mice (and the respective WT ones) represented three different stages in amyloid beta pathogenesis: (i) 3 mo old (pre-symptomatic stage), (ii) 6 mo old (start of symptoms, active neurodegeneration phase), and (iii) 12 mo old (terminal stage, extensive brain atrophy) (Fig 3A).

**Figure S3.**
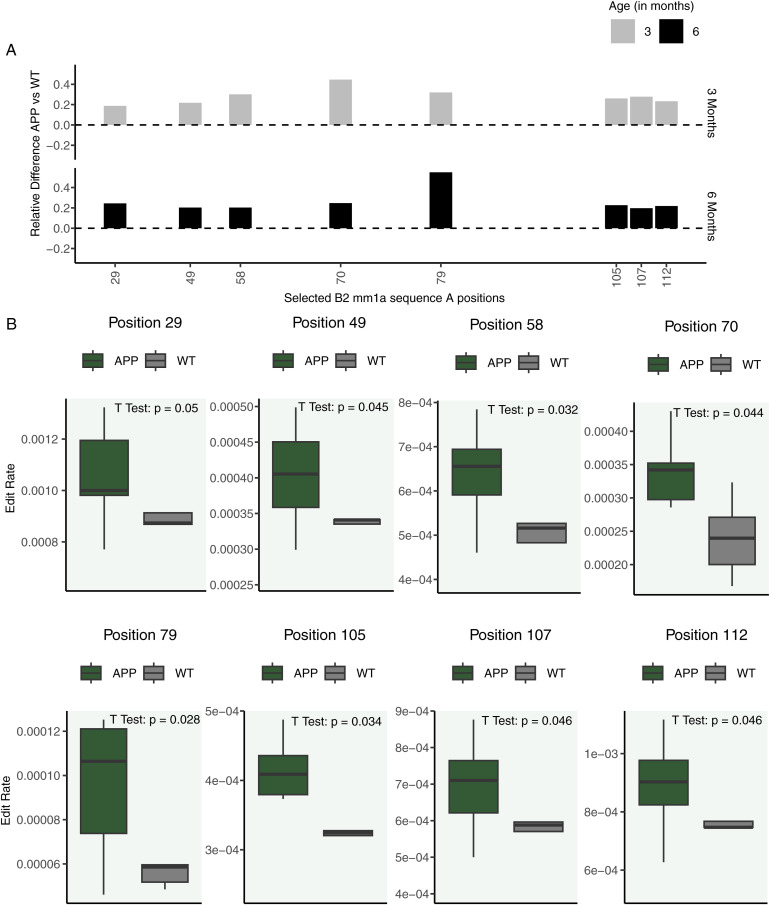
Site specific editing changes between APP and WT mice at 3 and 6 mo. **(A)** Relative differences in edit rate at selected subset positions in 3- and 6-mo-old mice (n = 3 for each treatment at each age group, except for 3-mo WT mice, which used n = 2). Positions were chosen from the Adar knockdown dataset (Fig 2C) according to two criteria: (i) they lie within the main body of the B2 element (approximately before position 120 nt) to avoid poly-A tail regions that may introduce artifacts, and (ii) they exhibit high editing magnitude in the knockdown data. Although all positions in Fig 2C correspond to adenosines in the consensus Mm1a sequence, editing magnitude varies markedly across sites. The subset shown here represents those positions with a relative edit rate > 0.0001 with a statistically significant difference in editing (see panel (B) below) and is used in all subsequent supplementary analyses (Figure is partially identical with Fig 3 upper two panels and presented here to enable comparison with panel (B) below). **(B)** Boxplots comparing the above subset-site editing between APP and WT mice at 3 and 6 mo. Both ages show elevated editing under disease conditions, consistent with Fig 3B. *P*-values were obtained by a one-tailed *t*-test. Box plots are produced without outliers.

As in the *Adar1* and *Adarb1* knockdown experiments (AntiAdar), we calculated the relative A-to-I edit rate at each B2 RNA position for each sample, and then the relative edit rate per position among samples within the same age and genotype group. Subsequently, to compare the A-to-I edit rate of all positions between APP and WT mice for each age group, we generated cumulative distribution plots of A-to-I editing across all B2 RNA positions containing adenosines (Fig 3B). The resulting distributions suggested an increase in the relative edit rate in APP mice at 3 and 6 mo compared with WT mice of the same age (KS < 0.0001 and KS < 0.01, respectively), but this increase was eliminated and reversed in 12-mo-old mice. As shown in Fig 3C, the identified B2 RNA positions with significant relative edit rates (>10^−4^) correspond to the same positions identified in our AntiAdar experiments in Fig 2C. However, not all of these positions were found to differ significantly between APP and WT mice at 3 and 6 mo. As shown in Fig S3A and B, eight positions showed significant differences, and the relative change in editing rate of these positions is plotted in Fig 3D. This analysis confirmed an increase in A-to-I edit rate in eight positions in 3- and 6-mo APP mice (Figs 3D and S3A and B), whereas differences in edit rate in the rest of the positions did not reach the statistical significance threshold in either in the both direction or one-direction comparison in 3- or 6-mo-old mice.

**Figure S4.**
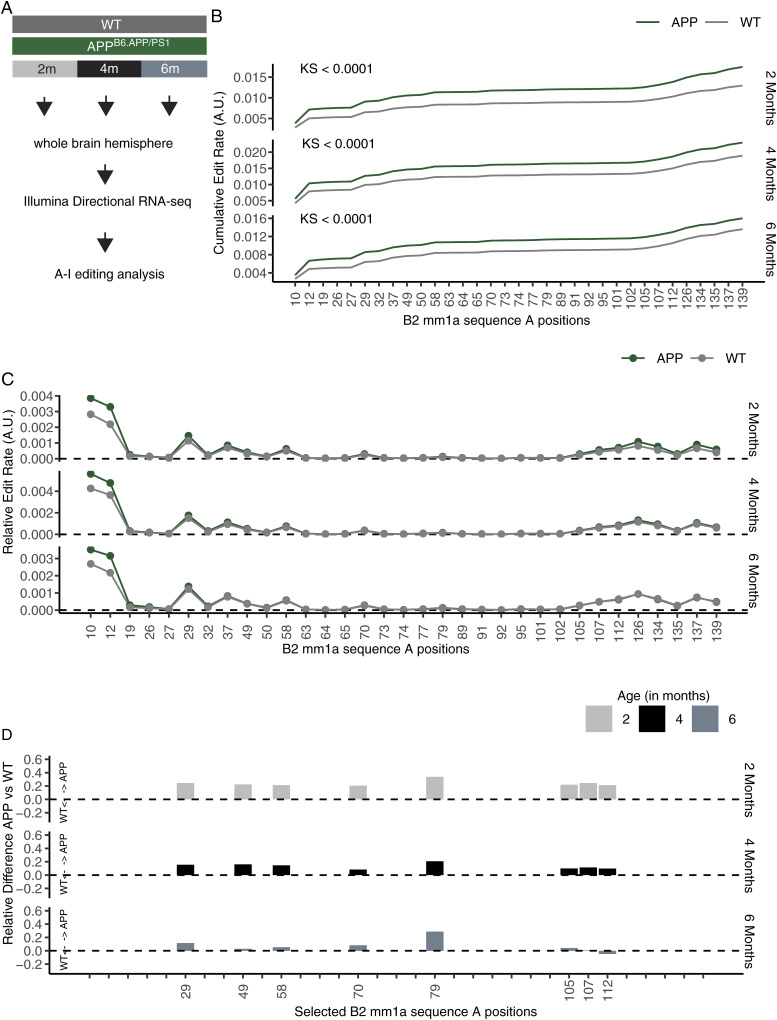
Validation of editing dynamics in an independent mouse model expressing human APP and PSEN1 transgenes (GEO GSE136861). This dataset includes whole-brain RNA-seq from APP and WT mice aged 2 (n = 10, 9, respectively), 4 (n = 5, 9, respectively), and 6 (n = 7, 13, respectively) months to match the primary dataset (Fig 3). The overall pattern parallels our observations in Fig 3: APP mice exhibit increased editing. **(A)** Schematic overview of the external dataset and processing workflow. **(B)** Cumulative distribution of edit rate across B2 RNAs (mm1a) per position in APP versus WT groups across the three age ranges. The Y-axis indicates mean cumulative editing rate; statistical values are from Kolmogorov–Smirnov tests. **(C)** Position-specific mean relative edit rates across all consensus A positions (AU, arbitrary units). **(D)** Relative editing differences (APP and WT) along the B2 Mm1a sequence, with positive values denoting higher editing in APP.

**Figure S5.**
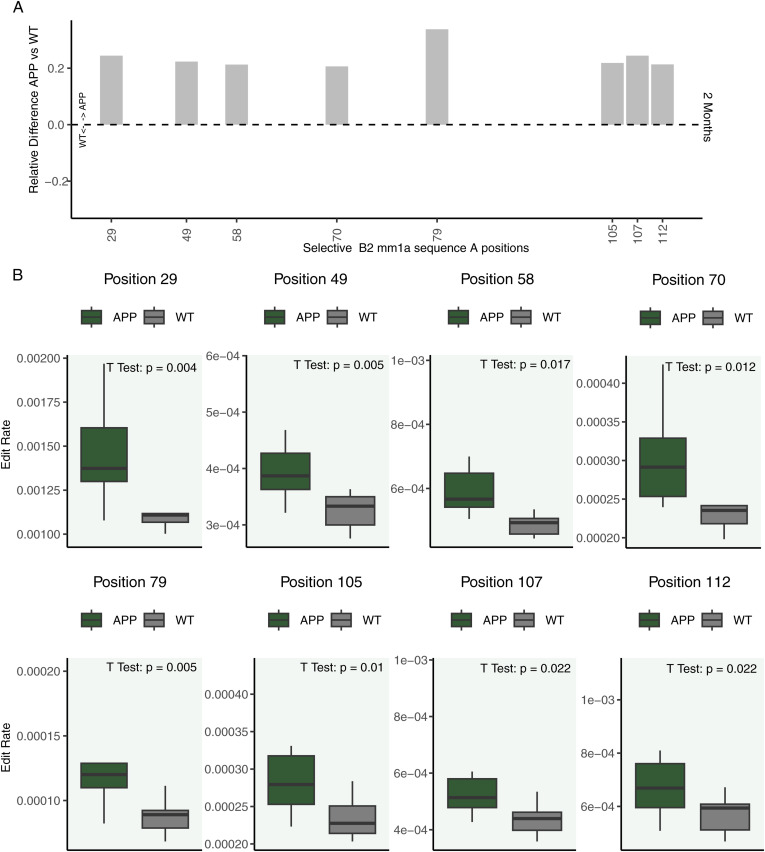
Site specific editing changes in 2-mo-old mice from the external dataset. **(A)** Relative editing differences at subset positions (as defined in Fig S3) in 2-mo-old mice from the external dataset (see Fig S4) (n = 10 and 9 for APP and WT, respectively). The figure is partially identical to Fig S4 upper panel and presented here to enable comparison with panel B below. **(B)** Boxplots comparing subset positions between APP and WT groups at 2 mo. *P*-values were obtained using one-tailed *t*-tests.

To validate our findings in an independent amyloid beta pathology system and a dataset not generated by our laboratory, we applied our customized SINE A-to-I editing analysis to publicly available brain transcriptome data from an additional mouse model of this pathology (APP^B6.APPP/PSI^). This model exhibits progressive amyloid beta accumulation similar to that observed in the APP^NL-G-F^ strain, but with distinct kinetics and genetic background and a predominant neuroinflammatory phenotype (Chintapaludi et al, 2020). Available Illumina RNA-seq data from whole brain tissue included both transgenic and wild-type control animals up to 6 months old (Fig S4A). As with the analysis of the APP^NL-G-F^ strain, we calculated the relative A-to-I edit rate at each B2 RNA position for every sample and averaged the values within each age and genotype group. Cumulative distributions of A-to-I editing across all adenosine-containing positions (Fig S4B) revealed a similar pattern to that observed in our mouse hippocampus dataset: editing levels were increased in the brains of transgenic mice with amyloid beta pathology of the same age (KS < 0.0001). The positions with significant relative edit rates (>10^−4^) (Fig S4C) overlapped with those detected in the APP^NL-G-F^ model (Fig S4D), with differences in relative edit rate between APP and WT mice of the eight positions identified in the APP^NL-G-F^ model also observed in the APP^B6.APPP/PSI^ model (Fig S5A and B). Importantly, this external dataset differs from our primary cohort in both tissue source and genetic background, comprising whole-brain RNA-seq data rather than hippocampus-specific samples and employing a transgenic APP/PSEN1 overexpression model as opposed to the APP knock-in strategy used here. The observation of consistent editing patterns across these distinct experimental settings supports the robustness of the identified A-to-I editing changes and demonstrates that the observed editing pattern is not model-specific or dataset-dependent but instead represents a consistent molecular feature of amyloid beta-associated neurodegeneration.

Overall, these data show that, during the first stages of amyloid beta pathology in mouse hippocampus and whole brain tissue, A-to-I editing at specific conserved positions in B2 RNAs is increased.

### Estimation of RNA modification rates using nanopore direct RNA-seq

To evaluate, using an independent method, the level of adenosine modifications estimated by our customized Illumina-based pipeline, we applied Oxford Nanopore Technologies direct RNA-seq, which enables direct measurement of RNA modifications from raw direct RNA-seq signal data. Using a previously described approach for signal-level analysis of non-standard (modified) bases in this type of data (Pratanwanich et al, 2021), we examined hippocampal RNA samples from both our AntiAdar and scramble siRNA-treated cells (Scramble), as well as from the APP^NL-G-F^ (APP) and WT 6-mo-old mice used in this study.

To ensure comparability with our Illumina editing rate analysis, nanopore reads were aligned to a unique B2-ome reference, in which identical B2 sequences across genomic loci were again collapsed into a single representative sequence. Raw nanopore current signals were first aggregated into 5-mer (k-mer) bins using nanopolish and subsequently analyzed with Xpore, a statistical model that estimates modification rates at each k-mer position. Only k-mers containing adenosine residues (the potential substrates for A-to-I editing) and with modification-rate values below 0.99 were retained, as higher extreme rates could represent artifacts of the model. In addition, to obtain the exact electric current signal distributions of modified versus non-modified adenosines in these samples, nanopore reads aligned to B2 loci were extracted, and the resulting reads within the B2-relative genomics coordinate system were used to generate nanopolish processed signal plots.

**Figure 4.**
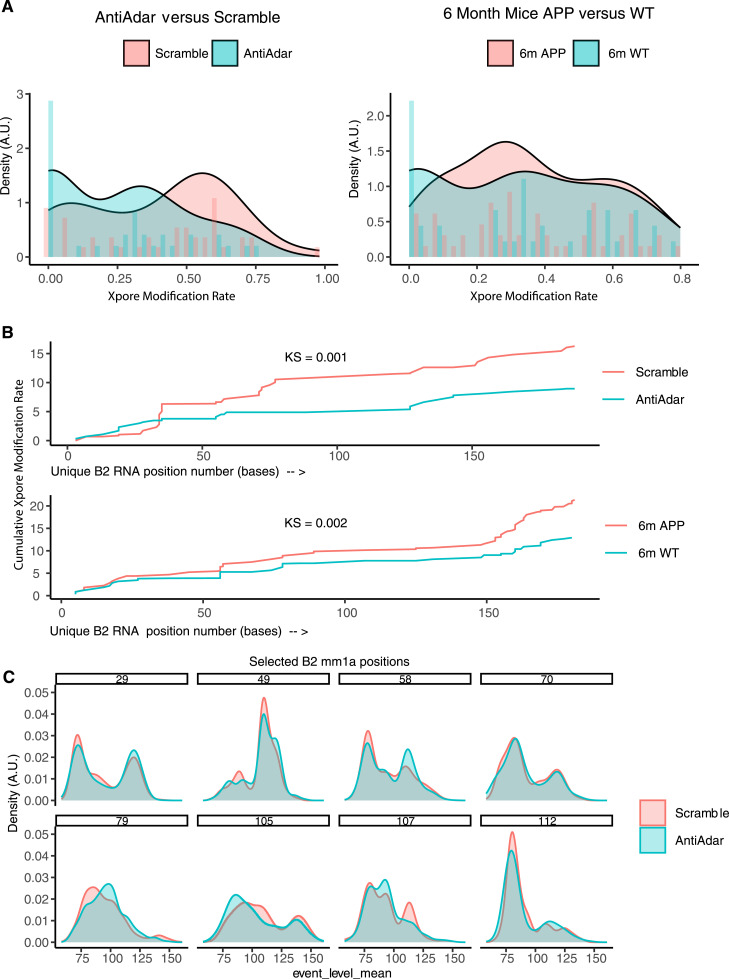
Nanopore direct RNA-seq signal analysis confirms observed RNA modifications in hippocampal cells and mouse hippocampi. **(A)** Distributions of Xpore-inferred modification rates across B2 RNA positions in AntiAdar versus Scramble HT-22 cells (RNA from four replicates per group pooled per run) and in 6-mo-old APP versus WT hippocampi (n = 3 per-sample group). Samples are concatenated group-wise. X-axis: modification-rate bins (overall range 0 [no editing] – 1 [100% editing]); Y-axis shows density for the curves and histograms, respectively, with histograms scaled down (¼ height) to better display the plot. Density is a smoothed measure of the number of points within a region, and so the points, when one sample group rises above another, indicate a higher proportion of genomic sites with modification rates within that range. **(B)** Cumulative modification-rate plots showing aggregate differences in editing across B2 RNAs (Kolmogorov–Smirnov test values). The X-axis represents position (in bases) relative to the B2 RNA start position for all read k-mers containing an adenosine. **(C)** Nanopolish signal-density plots at representative B2 positions previously identified as significantly edited (Fig 3D), illustrating group-specific raw-signal differences underlying Xpore estimates of panel (A). Nanopolish has been used to aggregate raw nanopore sequencing data into a k-mer-level format, which was then used as input to Xpore (see panel (A)) to estimate the modification rate for a comparison within a single genomic site and k-mer combination. The nanopolish plots displayed here visualize the raw-signal distributions that inform the Xpore statistical model of panel (A). Any observed differences between the Scramble and AntiAdar distributions correspond to differences in the proportions of non-standard bases (i.e., modified bases such as inosines) between the different samples tested, whereas identical distributions (as in position 70) would correspond to non-detectable changes in RNA modifications in that position.

Consistent with our Illumina-based findings, the Xpore-computed modification-rate distributions (Fig 4A) and cumulative modification-rate plots (Fig 4B) showed that the frequency of non-standard A base signals was higher in scramble-treated cells (Scramble) than after ADAR activity knockdown (AntiAdar) (KS = 0.001), and higher in 6-mo-old APP mice than in age-matched WT controls (KS = 0.002). This trend mirrors the increased A-to-I editing rates observed in our Illumina analysis. At a position-specific level, analysis of the raw nanopore signal distribution across the eight positions previously identified as significantly edited revealed clear differences in the signal profiles between experimental groups (Fig 4C) in all but one of these positions (position 70). These results further support the ability of our customized Illumina-based analysis pipeline in identifying modified adenosine sites within SINE B2 RNAs in our context.

### B2 RNA editing is increased in hippocampal cells in response to amyloid beta toxicity

**Figure 5.**
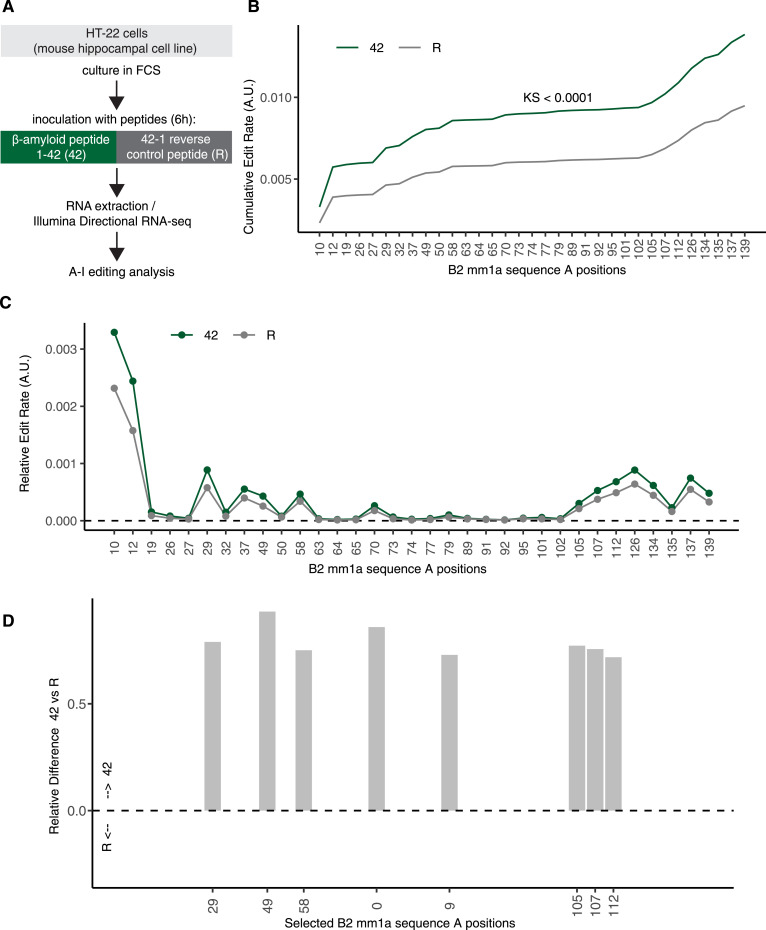
Acute amyloid beta exposure increases A-to-I editing of B2 RNAs in hippocampal cells. **(A)** Experimental outline of HT-22 cells treated for 6 h with amyloid-β_42_ peptides (42) or control reverse peptides (R) (n = 4) from transcriptome data from reference Cheng et al (2020). **(B)** Cumulative distribution of edit rate across B2 RNAs (mm1a) (Kolmogorov–Smirnov test value) (AU, arbitrary units). **(C)** Position-specific mean relative editing rates across all consensus A positions (AU, arbitrary units). **(D)** Relative differences in edit rate (42 – R) at each position; positive values denote increased editing under amyloid beta toxicity.

In our previous study, we had used a cellular model of amyloid beta toxicity that included inoculation of a hippocampal cell line (HT-22 cells) with amyloid beta peptides (1–42 peptides) (42) and control peptides (reverse 42-1) (R) for 6 h (Fig 5A) to test amyloid beta peptide immediate toxicity effects. Thus, we tested whether inoculation of HT-22 cells with these peptides could have effects on A-to-I editing levels of B2 RNAs similar to those observed in vivo.

**Figure S6.**
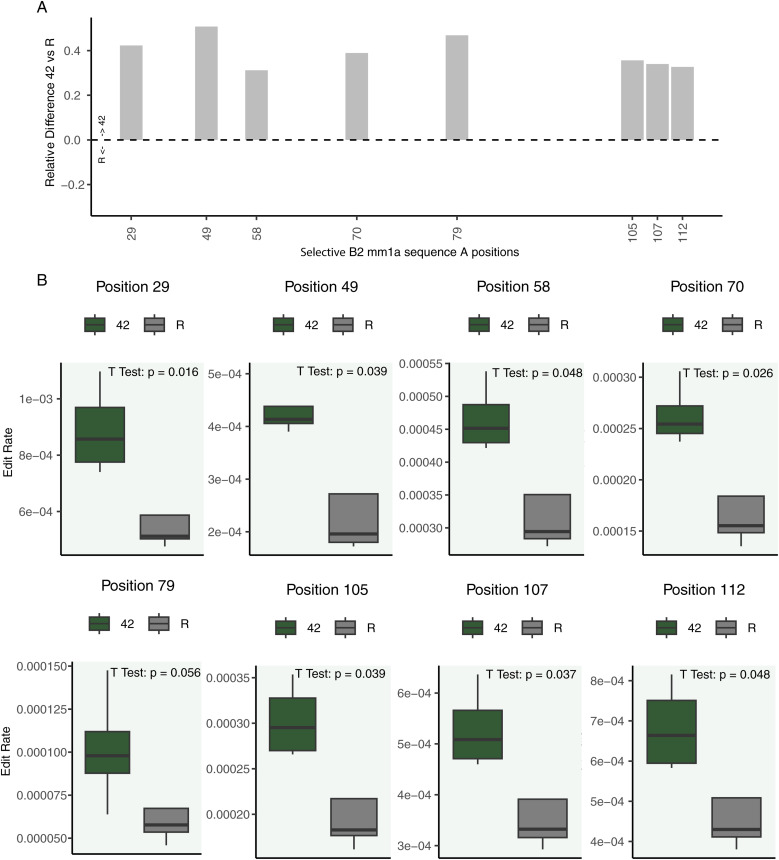
Subset-site editing changes in HT-22 cells exposed to amyloid-β toxicity (see Fig 5). **(A)** Relative editing differences at subset positions. Fig S3A legend describes how positions have been selected. (Figure is identical to Fig 5C upper and presented here to enable comparison with panel (B) below). **(B)** Boxplots comparing amyloid-treated (42) and reverse-peptide–treated (R) cells. *P*-values are from one-tailed *t*-tests.

As shown in the edit rate cumulative distributions of Fig 5B, amyloid beta peptides (42) induced a significant increase in total A-to-I editing across B2 RNAs compared with control peptide–treated cells (R) (KS < 0.0001). To determine whether this change reflected specific editing hotspots rather than a global shift, we calculated the relative editing rate at each B2 RNA position. The resulting position-specific plots (Figs 5C and D and S6A and B) revealed that the editing increase was in alignment with that observed at the same loci identified in AntiAdar and APP^NL-G-F^ versus WT analyses. In particular, the direction and statistical significance of these changes (Fig S6A and B) mirrored those observed in hippocampal tissue from 3- and 6-mo APP mice (Fig S3A and B), further supporting the consistency of increased edit rate across in vivo and in vitro models.

Taken together, these results indicate that acute amyloid beta exposure is sufficient to trigger an increase in A-to-I editing of B2 RNAs, recapitulating the editing pattern observed during the early stages of amyloid β pathology in vivo.

### *Adar/Adarb1* levels are increased in response to amyloid beta toxicity and pathology in hippocampal cells

**Figure 6.**
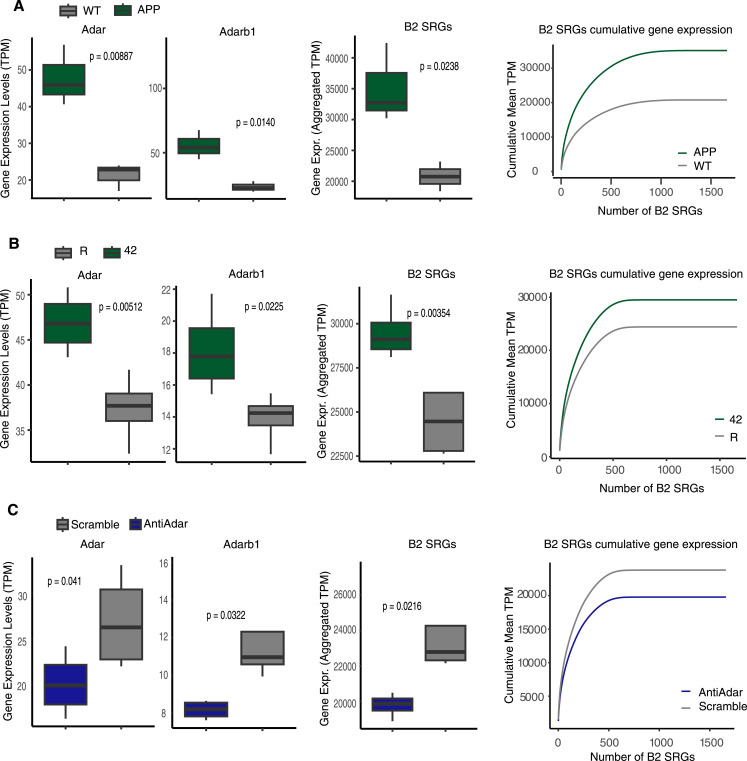
*Adar/Adarb1* expression and B2 stress-response gene levels parallel editing dynamics across models. The first two boxplots in each panel show the expression levels of *Adar* and *Adarb1*. The third boxplot shows the aggregate expression (in TPM) of B2 SRGs (1,684 genes). The cumulative expression (mean TPM) plot demonstrates that B2 SRG expression follows a continuous distribution not dominated by single transcripts. Expression is in TPM, aggregate TPM, or cumulative TPM as indicated (*P*-values, directional *t*-test). **(A)** Expression levels in 6-mo-old APP versus WT hippocampi (n = 3) of Fig 3. **(B)** Expression levels in HT-22 cells treated with amyloid beta peptides (42) or a control peptide (R) (n = 4) of Fig 5. **(C)** Expression levels in AntiAdar versus Scramble-treated HT-22 cells (n = 4) of Fig 2.

**Figure S7.**
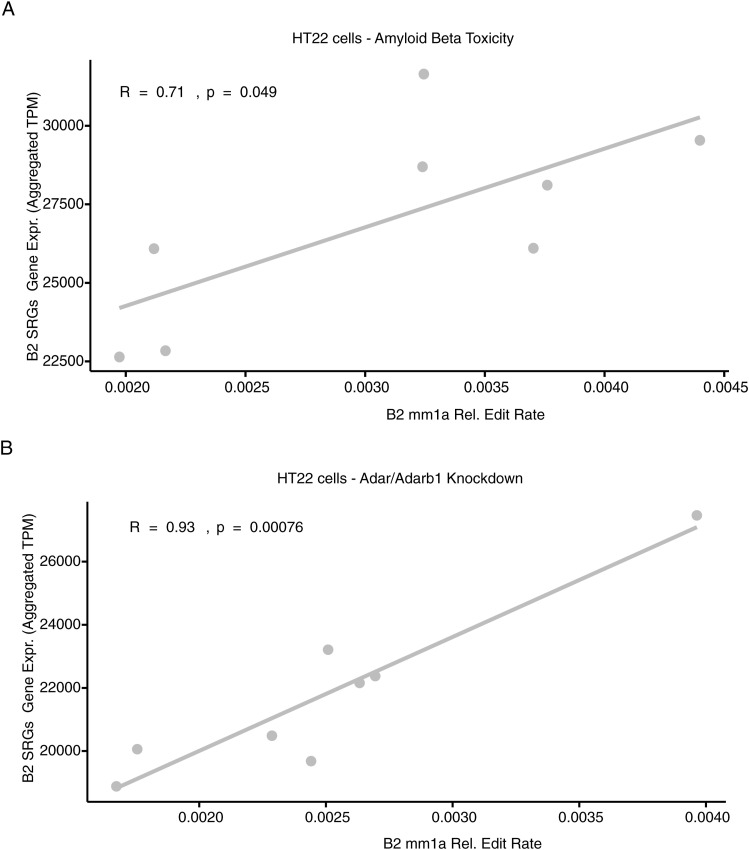
B2 SRGs expression and computed editing rate correlation. **(A, B)** Sample-wise correlation between B2 SRG expression and A-to-I editing in (A) during response to amyloid beta toxicity (Fig 5 dataset) and (B) siRNA-knockdown (Fig 2 dataset) in HT-22 cells, showing per replicate Pearson correlations between cumulative B2 SRG expression and total editing at the subset positions defined in Fig S3 (Panel (A): R = 0.71, *P* = 0.0049; Panel (B): R = 0.93, *P* = 0.00076). The positive correlations across both experiments support a tight coupling between SINE-derived gene activity and A-to-I editing levels in HT-22 cells.

We next examined whether the changes observed in B2 RNAs A-to-I editing were associated with similar alterations in *Adar/Adarb1* expression levels. In hippocampal tissues from APP mice, both *Adar and Adarb1* expression levels were found to be elevated, and this increase was accompanied by higher B2 SRG levels (Fig 6A). We then asked whether similar relationships could be observed in vitro. Indeed, in HT-22 hippocampal cells exposed to amyloid beta toxicity, both *Adar* and *Adarb1* transcript levels were also significantly increased, consistent with the elevated A-to-I editing levels detected in these cells, whereas the expression levels of B2 SINE RNA-regulated genes (B2 SRGs) were also found to be increased under amyloid beta toxicity conditions (Fig 6B). These changes aligned with changes in B2 RNA editing levels (Fig S7A).

The above findings raised the question whether reducing *Adar/Adarb1* expression could have a suppressive effect on B2 SRG levels, and to investigate this, we checked B2 SRG levels during siRNA-mediated *Adar/Adarb1* knockdown in HT-22 cells (AntiAdar) and the subsequent reduction in A-to-I editing levels (Fig S1). As shown in Fig 6C, B2 SRGs transcript levels were significantly lower after *AntiAdar* treatment than in scramble controls, confirming that decreased ADAR activity suppresses B2 SRGs up-regulation. These changes also aligned with changes in B2 RNA editing levels (Fig S7B).

Together, these results establish a clear connection between *Adar/Adarb1* expression, amyloid beta toxicity, and B2 SRG activation, suggesting that the ADAR A-to-I editing machinery may play a regulatory role in B2 RNA–mediated regulation of gene expression in hippocampal cells during amyloid beta toxicity.

## Discussion

Long considered to be products of “junk” DNA and transcriptional noise, SINE RNAs have been recently implicated in the cellular response to stress, and at the same time, designated as primary targets of the A-to-I editing machinery in the cell. This is the first study to identify specific positions within SINE RNAs that act as hotspots of A-to-I editing in the hippocampus and related amyloid beta toxicity models rather than showing only overall bulk changes across their length. This became possible through our novel analysis strategy, which assesses A-to-I editing of SINE RNAs by approaching these transcripts as a common population of RNA sequences produced by different genomic elements (mapping to the REPome) rather than as a set of separate genomic loci (mapping to the genome). Using this approach, we were able to describe distinct position-specific changes in the SINE RNA A-to-I epitranscriptome in response to amyloid beta pathology and toxicity in hippocampal cells and reveal that editing at specific B2 RNA positions correlates with changes in expression of B2 stress-response genes (B2 SRGs). Given the recently identified role of B2 RNA destabilization in the regulation of gene expression and response to cellular stress, these findings unveil a novel link among A-to-I editing, ADAR1 and ADAR2 enzymes, and B2 RNA–regulated gene expression during amyloid beta pathology.

Interestingly, our results demonstrate that editing of SINE RNAs occurs at discrete positions that are reproducible across independent datasets and experimental systems. Instead, we had expected a broadly distributed editing pattern across the entire length of SINE B2 RNAs based on a previously proposed SINE RNA editing mechanism that involves the formation of double-stranded RNA molecules from inverted SINE transcripts (Eisenberg & Levanon, 2018; Mehdipour et al, 2020). This new finding raises the potential that intramolecular double-strand RNA formations within the SINE B2 RNA that have been previously described in case of the B2 consensus sequence (Espinoza et al, 2007; Podbevsek et al, 2018; Sharma et al, 2024) may also play a role in the editing process beyond the mechanism of inverted SINE transcripts. However, a precise structural assignment would require dedicated future structural analysis of multiple different B2 RNAs because structural predictions based only on the consensus sequence do not fully capture the variability present in the underlying RNA population, which limits the interpretability of direct structure–editing overlays.

Using our REPome-based approach, we detected widespread deregulation of B2 RNA editing in amyloid beta pathology, which would have remained undetected by standard genome-based methods. This deregulation was observed both in vivo, in mouse hippocampi and brains affected by amyloid beta pathology, and in vitro, in hippocampal cells exposed to amyloid beta toxicity. Importantly, the early increase in B2 RNA editing coincided with elevated *Adar/Adarb1* expression levels, suggesting that the ADAR-mediated editing machinery is part of an early epitranscriptomic response to amyloid beta toxicity. Although increased editing is observed at early stages of pathology, it should be noted that the 3-mo WT group includes a limited number of samples (n = 2), which reduces statistical power for this comparison. Therefore, although the observed trend is consistent with findings at later time points and across independent datasets, the results at this pre-symptomatic stage should be interpreted with appropriate caution.

**Figure S8.**
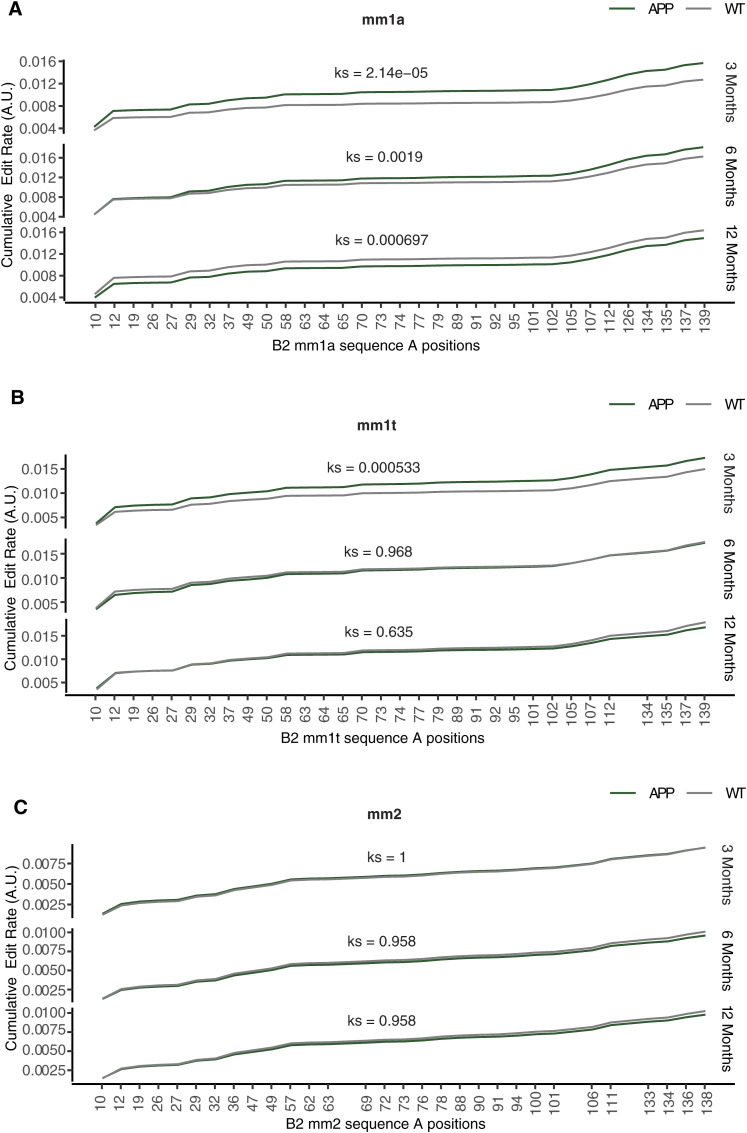
Cumulative edit rate between APP and WT samples (as described in Fig 3B) across three different B2 RNA subfamilies. **(A)** Cumulative edit rate specific to B2 mm1a subfamily. **(B)** Cumulative edit rate specific to B2 mm1t subfamily. **(C)** Cumulative edit rate specific to B2 mm2 subfamily.

In this study, we focused on the mm1a B2 RNA subfamily, as mm1a—and the closely related mm1t family—most closely match the canonical B2 RNA consensus used in previous B2 RNA functional studies. This ensures that positional editing measurements remain biologically comparable and meaningful because mm1a elements retain the conserved motifs required for both B2 ribozyme activity and RNA Pol II suppression. In this context, it should be noted that position-specific editing analyses presented in the main figures are restricted to reads mapping to the mm1a subfamily, which most closely matches the canonical B2 consensus and allows consistent positional interpretation. Importantly, however, different B2 subfamilies show distinct expression and editing patterns. As illustrated in Fig S8A–C, in the case of amyloid beta pathology in mouse models tested in this study, although mm1t editing profiles align with those of mm1a, mm2 elements—harboring several insertions and deletions compared with the previous two subfamilies—display a divergent pattern. These sequence differences include the absence of several conserved motifs present in the canonical B2 consensus, which may contribute to altered RNA secondary structure formation and consequently distinct editing behaviors. These observations indicate that subfamily-specific sequence architectures very likely shape editing behavior, highlighting the need for future functional studies to explore further subfamily-resolved SINE RNA editing.

Our findings raise several new questions. One key issue is whether A-to-I editing serves a protective role—stabilizing B2 RNAs and mitigating their overprocessing—or whether it facilitates B2 RNA turnover and contributes to stress-induced transcriptome remodeling.

Distinguishing among these possibilities will require direct experimental modulation of ADAR activity and editing levels in cellular stress-response systems. The coordinated up-regulation of *Adar/Adarb1* that we observed in amyloid beta–exposed hippocampal cells suggests a concerted response of the editing machinery, potentially aimed at modulating the stability and regulatory potential of B2 RNAs under cellular stress. Although both ADAR1 and ADAR2 are known to contribute to A-to-I editing, the present study does not distinguish enzyme-specific targeting. Our use of combined *Adar*/*Adarb1* knockdown was designed to ensure a robust reduction in overall editing and avoid potential compensatory effects between the two enzymes. Dissecting ADAR1-versus ADAR2-specific contributions to B2 RNA editing remains an important direction for future work. Future studies should also examine whether ADAR1 and ADAR2 act redundantly or perform distinct editing functions on B2 RNAs, given their differential subcellular localization and substrate preferences. ADAR2’s role in protein recoding of genes like *Gria2* is well accepted, whereas ADAR1 more prominently suppresses innate immunity; however, at the same time, both enzymes demonstrably can edit B2 RNAs, though with potentially different efficiencies (Chalk et al, 2019). Another important question concerns the mechanistic interplay between RNA editing and the intrinsic ribozyme activity of B2 RNAs. Editing events occurring near or within self-cleavage sites could directly affect RNA stability by altering secondary structures critical for B2 RNA processing. Understanding how editing influences these structural and functional properties will provide insight into how SINE RNAs contribute to cellular resilience or vulnerability during neurodegeneration. Finally, although nanopore direct RNA-seq provided independent support of editing changes detected by short-read sequencing, the type of nanopore flowcell used cannot unambiguously distinguish inosine from other RNA modifications. Therefore, it cannot be excluded that part of the detected signal variation reflects additional, non-inosine base modifications or potential crosstalk between differing base moieties, which could also partially explain the discrepancy between the illumina and nanopore results at position 70. For instance, in mRNAs, a growing body of evidence suggests that m6A sites and A-to-I editing events are negatively correlated (Xiang et al, 2018), and although B2 RNAs are not known to host m6A moieties, other potential modifications may be impacted by a general loss of ADAR activity. Moreover, the fact that position 70 was identified as significantly edited by the illumina-based analysis did not show a corresponding separation in nanopore signal distributions may reflect limitations of the R9.4.1 nanopore chemistry and associated signal models, as modification detectability depends on local sequence and k-mer context, whereas local read coverage or signal perturbation at this site may also have limited discrimination power.

**Figure S9.**
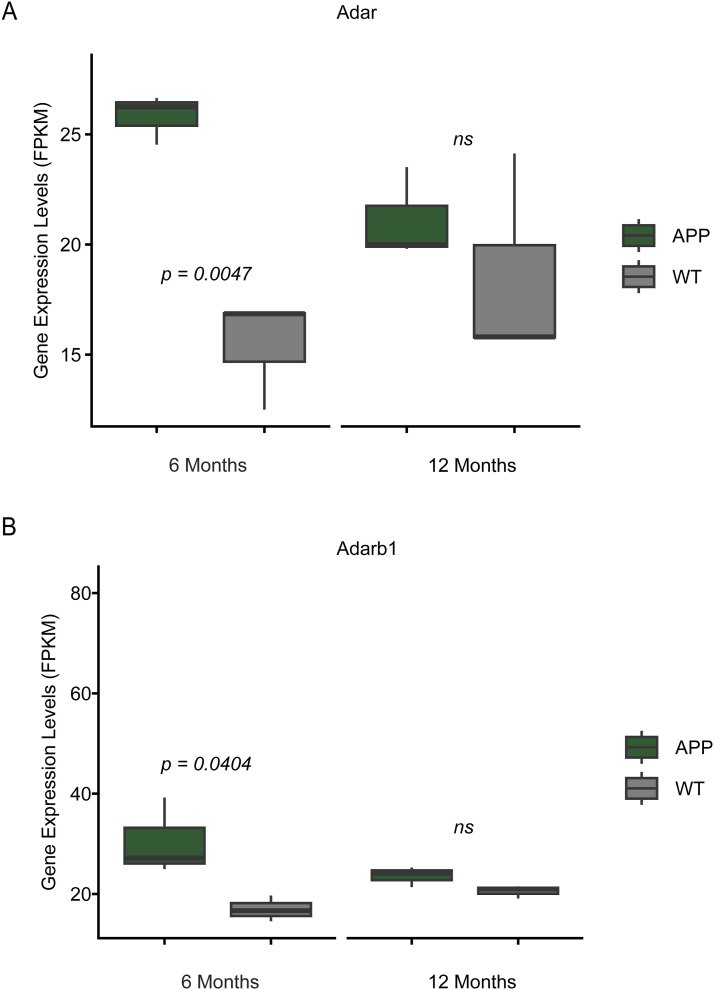
*Adar* and *Adar1b* expression levels in 6- and 12-mo old APP and WT mice. **(A)** Expression levels of *Adar* in 6- and 12-mo-old APP versus WT hippocampi of Fig 3. Expression is in FPKM as indicated (n = 3, directional *t*-test). **(B)** Expression levels of *Adarb1* in 6- and 12-mo-old APP versus WT hippocampi of Fig 3. Expression is in FPKM as indicated (n = 3, directional *t*-test).

Overall, elucidating factors, such as ADAR activity, that are upstream of SINE RNA A-to-I editing may provide additional insights regarding the mechanisms underlying the role of SINE RNAs in cell function and related molecular pathologies such as neurodegeneration. For example, changes in ADAR expression are likely contributing to the observed increase in B2 RNA editing in APP mice relative to WT mice at 6 mo, a difference that is no longer observed at 12 mo. As shown in Fig S9A and B, *Adar* and *Adarb1* expression levels are elevated in APP mice compared with WT at 6 mo, whereas this difference between genotypes is no longer present at 12 mo. This pattern mirrors the editing data, where higher A-to-I editing in APP mice relative to WT at 6 mo is lost at 12 mo. Together, these observations suggest that changes in ADAR expression may contribute, at least in part, to the stage-specific differences in B2 RNA editing between APP and WT animals, although, as mentioned above, more targeted studies are required to dissect any distinct roles of *Adar* and *Adarb1* in this. This would further advance our understanding of SINE RNAs as potential novel therapeutic targets and not just transcriptional noise. It is notable that *Adarb1* (ADAR2) and *Adarb2* (ADAR3) genes are, in fact, B2 RNA–regulated stress-response genes themselves (Cheng et al, 2020), whereas Adarb1 transcription is amyloid-responsive (Cheng et al, 2020). By highlighting the site-specific A-to-I editing events in B2 RNAs, our study uncovers the initial results towards a more complete understanding of the complex interacting regulatory axis of A-to-I editing and B2 RNA regulation of stress and amyloid beta toxicity. The human B2 RNA ortholog, SINE Alu RNA (entirely different sequence and structure, but seemingly hosts similar cellular interactions) is also potentially differentially edited in Alzheimer’s disease patients’ hippocampal vasculature (Crooke et al, 2022). Relatedly, we previously found that SINE Alu RNAs are increasingly processed in Alzheimer’s disease prefrontal cortex and hippocampus samples, which is likely linked to widespread changes in gene expression, relative to age-matched healthy brain tissue (Cheng et al, 2021). Therefore, how the overarching regulatory axis of ADAR regulation and SINE RNA biochemistry interacts will also be of key importance to furthering our understanding of neurodegenerative diseases such as Alzheimer’s disease.

## Materials and Methods

### Cell culture and molecular assays

HT-22 cells were cultured as described previously (Cheng et al, 2020). Transfections of siRNAs against *Adar* and *Adarb1* siRNAs (IDT, see below) were performed through the generation of an equimolar siRNA pool at a10 nM final concentration as described before (Cheng et al, 2020) with transfections for 36 h before harvesting for RNA extraction. For the EndoV assays, 250 ng total RNA was incubated with 5 µl 10X NEB 4 buffer and 1 µl EndoV enzyme (M0305S; NEB) in a 50 µl reaction for 1 h at room temperature. Untreated samples were topped up with 1 µl of nuclease-free water. Reverse transcription quantitative PCR (RT-qPCR) was performed as described before (Cheng et al, 2020) using the same B2 RNA primers. *Adar* primers used were Forward: 5′-GCCAAAGACAGTGGTCAACCAG-3′; Reverse: 5′-GAACAAGGATGTTGCTGAGGAGC-3′. *Adarb1* primers used were Forward: 5′–GTATGACGCCAGACTCTCACCA-3′; Reverse: 5′-CAGGTCTGGATGCTGGCATTTG-3′. For normalization of RT-qPCR results, in the case of B2 RNA analysis, we used mouse 7SK RNA with primers Forward 5′-GACATCTGTCACCCCATTGA-3′, Reverse 5′-GCCTCATTTGGATGTGTCTG-3′. In the case of *Adar* and *Adarb1*, we used *Hprt* with primers Forward 5′-TCCTCCTCAGACCGCTTTT-3′ and Reverse 5′-CCTGGTTCATCATCGCTAATC-3′. IDT siRNAs used in this study are as follows: mm.Ri.Adarb1.13.1 or DsiRNA 5′-AAUCUGAGUCUUUCAGCAUUCUGAC-3′ 3′-ACUUAGACUCAGAAAGUCGUAAAGACUG-5′, mm.Ri.Adar13.1 or DsiRNA 5′-UCAGUGUUUAUGAUUCCAAAAGACA-3′ 3′-UCAGUCACAAAUACUAAGGUUUUCUGU-5′.

### Nanopore direct RNA-seq and analysis

In the case of HT-22 cells of the AntiAdar experiments, RNA was pooled from n = 4 replicate sets before sequencing for each sample type. The libraries were prepared according to the vendor-recommended protocol for the SQK_RNA002 direct RNA-seq kit using 1 μg of starting material. Briefly, 9.5 μl of RNA was mixed with 1 μl RTA adaptor (Nanopore), 1.5 μl T4 DNA ligase (M0202M; NEB), and 3 μl NEBNext quick ligation buffer (B6058S; NEB), and incubated for 15 min at room temperature. The first-strand synthesis reaction was constructed from the ligation product by adding 9 μl H2O, 2 μl dNTP mix (N0447S; NEB), 8 μl FS buffer (18080093; Invitrogen), 4 μl 0.1 M DTT (18080093; Invitrogen), and 2 μl Superscript III reverse transcriptase (18080093; Invitrogen) and incubated at 50°C for 50 min, 70°C for 10 min, then placed immediately on ice. The sample was then subjected to a 1.8× bead cleanup using the vendor-recommended protocol with Mag-Bind TotalPure NGS beads (M1378; Omega Bio-Tek), eluting in 23 μl H2O. 6 μl of the RMX adaptor (Nanopore), 8 μl of the NEBNext quick ligation buffer, and 3 μl of T4 DNA ligase were added to the eluted RNA:DNA hybrid and incubated for another 15 min at room temperature, followed by a 1× bead cleaning reaction. The prepared libraries were loaded and sequenced on an R9.4.1 PromethION flow cell using a PromethION 24 Beta instrument as per the manufacturer’s suggestions. These libraries were base called using Guppy version 3.2.10.

In the case of mice hippocampi, 6-mo-old APP and WT mouse samples were sequenced individually for each replicate using our previously published NERD-seq methodology (Saville et al, 2024). Briefly, 1.5 μg total RNA was size selected using the Invitrogen MirVana kit (AM1561); 90 μl of the short elution was polyadenylated using 12 μl 10× polyA polymerase buffer (B0276; NEB), 12 μl 10 mM ATP (B0756A; NEB), 6 μl polyA polymerase (M0276; NEB), and incubated at 37°C for 30 min. The polyadenylated short fraction and the long fraction were combined, purified, and concentrated using the RNeasy MinElute kit (74204; QIAGEN). 12 μl of the RNA was adaptor ligated using 4 μl NEBNext Quick ligation buffer (B6058), 0.66 μl RNA CS (Nanopore), 1.3 μl RTA adaptor (Nanopore), 2 μl 2,000,000 U T4 DNA ligase (M0202; NEB). The reaction was incubated at room temperature for 10 min. The sample was subsequently reverse transcribed using 1.5 μl of the Lucigen OmniAmp polymerase (F831942-1), 10 μl 10× OmniAmp buffer (F883707-1; Lucigen), 8 μl dNTP (N0447; NEB), 6 μl 100 mM MgSO4 (F98695-1; Lucigen), 10 μl Betaine (F881901-1; Lucigen), 5 μl random primer mix (S1330; NEB), and 39.5 μl RNase free H_2_O. The mixture was incubated at 50°C for 10 min and at 70°C for 2 min. Samples were cleaned with 2.88× Omega Mag-Bind TotalPure NGS beads. The 20 μl library was ligated to the Nanopore RMX adaptor using 6 μl RMX adaptor (Nanopore), 8 μl NEBNext quick ligation buffer, 3 μl RNase-free H2O, and 3 μl 2,000,000 U T4 DNA ligase and incubated for 10 min at room temperature. The adapted library was 1x bead cleaned using the Omega Mag-Bind TotalPure NGS beads, eluted in nanopore elution buffer. The library was loaded onto the nanopore PromethION or MinION according to manufacturer’s instructions. These libraries were base called using Guppy version 3.0.

Samples were pooled group-wise before mapping. Reads were mapped using minimap2 version 2.24 with the following parameters: -ax sr -uf --secondary = yes. Resulting BAM files were then sorted and indexed using SAMtools-1.2.1. Eventalign tables were generated using nanopolish 0.14.0 (Loman et al, 2015) and modrates were measured using Xpore 2.0 (Pratanwanich et al, 2021) where nanopolish is a prerequisite for Xpore. Xpore arranges raw-signal data from nanopore sequencing by matching sequence k-mer and mapping position. We filter these data by removing modification-rate values greater than 0.99 (assumed to be an artifact of Xpore’s model because a 100% editing rate should not occur biologically); we also filter for k-mer positions containing an A to give a suitable substrate for A-to-I editing, and finally, we filter to remove absolute value of differential modification rate less than 0.2 to ensure only strongly modified sites remain. These data are used to produce the Xpore signal plots in Fig 4A and B. To produce raw-signal plots (Fig 4C), data were aligned using minimap2 with the same version and parameters on ensemble release 102 *Mus musculus* genome (mm10). SAMtools was used as previously described to sort and index the BAM files. Nanopolish 2.1 was run on these BAM files. Then the coordinate system from these nanopolish tables was intersected with the coordinates of B2 Mm1a in the genome using bedtools v2.31.1 with the -wo flag (Quinlan & Hall, 2010). The resulting nanopolish table, now only containing nanopolish signal, which intersects B2 loci, was used to generate raw-signal plots in Fig 4C.

### Bioinformatics analysis of SINE RNA A-to-I editing

A custom reference was constructed from unique B2 elements in the mouse genome, hence called the REPome (unique B2-ome). For the construction of the “REPome,” we retrieved B2 sequences for all identified B2 repeats (mm1a, mm1t, and mm2) within the range of 125 bp or longer to exclude truncated forms from UCSC mm10 Repeat Masker (August 2018) and subsequently collapsed sequences present in our list more than once into one unique representative sequence, resulting in only unique B2 sequences. RNA-seq reads for each sample were mapped to the REPome using bwa-0.7.19 (Li et al, 2009) with the following parameters: aln, with a maximum of 10 allowed mismatches. The goal of aligning to the unique REPome was to ensure that each element is present only once in the reference. After alignment, files were converted to BAM format, sorted, and indexed using SAMtools-1.21 (Li et al, 2009). Then, the sense-aligning fraction was extracted from the SAMfile (SAMtools flag -F 16). Subsequently, the RNA edit detection software REDItools v1.2.1 (denovo script) (Picardi & Pesole, 2013) was used to produce tables of base alignments for each file. This software was run using the following modified parameters: -c 1, -q 1, -m 1, -O 0, -V 1, -n 0. The rational for the selection of these parameters was as follows; -c denotes the minimum read coverage, which was set to 1 as alignment on the unique reference may produce sparse read coverage; -q denotes minimum quality score, which was set to 1 as highly edited RNA may appear to the sequencer as low quality; -m is mapping quality score with the same rationale as previous; -O is min homopolymer length, which was set to zero so that REDItools will treat homopolymeric regions of the read identically to the heterogeneous regions; -n is the minimum editing frequency, set to 0 for maximum sensitivity.

After the REDItools tables were produced, we merged them with file identifiers into one single table. Then we computed the editing rate as follows. Initially, we constructed two sets of proportion statistics from base frequencies. Within each sample and for each position (1–180) of the B2 RNAs, we computed the counts of all reference A, T, C, and G, respectively, terming these “expected” counts. We repeated this count for all bases that are present in the reads, terming these “observed” counts. These counts are normalized to the sum of all expected and observed bases, respectively, yielding proportions. Thus, for each sample and B2 position, we generated two sets of proportion statistics, each of which was composed of four proportions, A, T, C, G (sum to 1). The first set was the “expected” proportions, which were generated from the expressed (>0 read coverage) regions of the unique B2-ome, without weighting the reference base contribution by the number of reads. The second set was the “observed” proportions, which were generated from the bulk aligned reads regardless of which sequence they aligned to. These proportions were used in the calculation of the editing rate per sample and position, which was (obsGprop/(obsGprop+obsAprop))/expA. This editing rate was based on the canonical G/(A+G); however, it used the population-level observed proportions previously described. Lastly, the editing rate was normalized to the number of unique reference regions, which have an A at the given position. REDItools parameters were selected to maximize recovery of aligned read information from repetitive B2 RNA regions. In this framework, REDItools was used exclusively to aggregate read and reference base composition data rather than to directly perform RNA editing calling. Consequently, the permissive parameter settings were applied to maximize sensitivity at the level of underlying alignment information. Although such settings could theoretically increase false-positive edit detection in low-coverage regions in conventional REDItools-based analyses, this issue is mitigated in our population-based framework because editing rates are computed independently from aggregated observed and expected base distributions across the REPome. Read-derived base proportions are calculated across all aligned reads, naturally weighting positions according to sequencing coverage, whereas reference-derived proportions are computed only from reference regions with detectable coverage. This approach minimizes the contribution of unsupported low-coverage positions while preserving sensitivity across repetitive SINE-derived transcripts.

In addition, we calculated this rate separately for each B2 subfamily, selected B2 Mm1a elements, and then we examined only positions within the B2 Mm1a consensus sequence that contain an A. In particular, REDItools tables generate base composition summaries for each unique REPome reference sequence, preserving B2 subfamily identity. For each position in each sample, observed and expected base proportions are calculated for mm1a, mm1t, and mm2 elements, and edit rates are computed independently for each subfamily. Analyses presented in the main figures focus on the mm1a-derived edit rates, using positions corresponding to adenosines in the mm1a consensus sequence. Importantly, alignment using bwa aln produces a single read-to-reference assignment, thereby avoiding cross-subfamily alignment ambiguity.

The editing rate in Fig S1C was calculated using the raw REDItools table. We filtered this table for positions that have an A in the reference and then computed the editing rate as G/(A+G), where these letters refer to the counts of reads aligning to that position. Then, we computed the mean of this edit rate across positions and samples to obtain a per-sample per-position rate in parity with our method.

*t* tests (where applicable) were based on directional hypotheses derived from the experimental design. In the siRNA-mediated *Adar*/*Adarb1* knockdown experiments, the directional hypothesis assumed reduced editing after ADAR depletion and, therefore, higher editing levels in the Scramble controls. Cumulative distribution comparisons were performed using two-sample Kolmogorov–Smirnov (KS) tests, which evaluate nondirectional differences between distributions.

### Gene expression analysis

B2 stress-response genes are genomic loci that have been shown to be regulated by B2 RNA. The list of gene names was retrieved from our previous study (Cheng et al, 2020). To calculate the gene expression levels, we first aligned fastq files against Ensembl release 102 Mus Musculus genome GRCm38 using Hisat2 v2.2.0 with the –dta option. The gene expression levels were calculated by using Stringtie 2.2.1 with -G, -A, -B, -e parameters in conjunction with transcriptome GTFs of the same Ensembl release. TPM levels from the analyzed gene or set of genes were extracted for each sample from the resulting abundance files.

## Supplementary Material

Reviewer comments

## Data Availability

Raw Illumina RNA-seq data (fastq files) for the APP/WT mouse and HT-22 transcriptome data originate from our previous study (Cheng et al, 2020) and are deposited in GEO with access number GSE149243. Immunohistochemistry and behavioral studies of this mouse model are described in our previous study (Mehla et al, 2019). The tested mice carry the Arctic, Swedish, and Beyreuther/Iberian mutations (APPNL-G-F/NL-G-F) and were provided by the RIKEN Center for Brain Science, Japan; the colony was maintained at the University of Lethbridge vivarium. The external mouse dataset comprises RNA-seq data from mice with human transgenes for mutant forms of APP and PSEN1, as well as WT controls. The dataset was retrieved from the GEO access number GSE136861 series. Novel RNA-seq data of this study have been deposited to GEO with access number GSE316378 for Illumina and nanopore RNA-seq of RNA from the HT-22 cells of the AntiAdar experiments and GSE171312 for nanopore RNA-seq of RNA from the mice hippocampi of the APPNL-G-F/NL-G-F mice mentioned above.
