# Supplementary material for: Differential A-to-I editing of SINE B2 RNAs unveils an epitranscriptome response to Aβ neurotoxicity
Source: Life Sci Alliance. 2026 Jun 16;9(9):e202603668. doi: 10.26508/lsa.202603668 (PMC13271943; doi:10.26508/lsa.202603668)
Supplement: Reviewer comments [file LSA-2026-03668_review_history.pdf]

Differential A to I editing of SINE B2 RNAs unveils an epitranscriptome response to Aβ neurotoxicity

Liam Mitchell, Luke Saville, Babita Gollen, Travis Haight, Riya Roy, Cody Turner, Yubo Cheng, Igor Kovalchuk, Majid Mohajerani, and Athanasios Zovoilis
DOI: https://doi.org/10.26508/lsa.202603668

Corresponding author(s): Athanasios Zovoilis, University of Manitoba

Review Timeline:

Submission Date:	2026-02-13
Editorial Decision:	2026-04-02
Revision Received:	2026-05-27
Accepted:	2026-06-01

Scientific Editor: Tim Fessenden

Transaction Report:

(Note: With the exception of the correction of typographical or spelling errors that could be a source of ambiguity, letters and reports are not edited. The original formatting of letters and referee reports may not be reflected in this compilation.)

Please note that the manuscript was reviewed at *Review Commons* and these reports were taken into account in the decision-making process at *Life Science Alliance.*

Review COMMONS

Reviews

Review #1

1. Evidence, reproducibility and clarity:

In the submitted manuscript, the authors explored ADAR-mediated A-to-I editing in B2 sine RNAs. They developed a computational approach to measure editing rates at a population level and found that editing at certain positions in the B2 RNA was dysregulated in mouse hippocampal cells suffering from amyloid beta toxicity. While the observations are interesting, the authors can potentially delve deeper into the underlying mechanisms. Below are some comments that I have for improving the manuscript.

Major comments:

1. After mapping the reads to the REPome, the authors calculated the proportions of base compositions within the whole population of B2 RNAs instead of calculating the RNA editing rate for each distinct member of the REPome. What advantage(s) are there to the authors' reported method vis-a-vis a more conventional approach of simply calculating the editing levels for each distinct B2 RNA "gene" (i.e. member of the REPome)?

2. Figure 2C: What is the p-value (WT vs APP) for 3m, 6m, and 12m?

3. Figure 4: Is the editing of position 49 increased in all members of the REPome or is the increase observed only in certain B2 RNAs? The location of the adenosine at position 49 (figure 3A) also appears to be non-ideal for ADAR-mediated editing. Are there any sequence variations in the B2 RNA that can produce a more stem-like structure around position 49? Perhaps these are the SINE RNAs whose editing is increased upon treatment with b-amyloid peptide.

4. Figure 5A-B: What are the p-values?

5. Figure 5C-F: (a) How do we know that 8-AzaA treatment was effective in blocking the activity of ADAR1? Can the authors check the editing levels of some known ADAR1 substrates in the control and treated cells? (b) 8-AzaA may have off-target effects in HT-22 cells. The authors should use an orthogonal method (e.g. siRNA knockdown) to confirm the results.

6. What happens when HT-22 cells are treated with both b-amyloid peptide and 8-AzaA? Will we see an even more pronounced amount of B2 RNA processing?

7. How does A-to-I editing confer a protective effect? Does editing prevent EZH2 from interacting with B2 RNA?

Minor comments:

1. In the introduction, the authors wrote that "multiple recent studies have revealed important functions for SINE genomic elements ... cryptic splicing sites and RNA circularization". No citations have been provided for the post-transcriptional functions (splicing and circularization). Please cite accordingly.

2. Typo on page 9: the authors wrote "A-to-I editing levels were found to correlate positively (r = 0.45) with full length B2 RNA levels (Fig.4A)". I think it should be Fig. 5A.

2. Significance:

Significance (Required)

The authors have provided some evidence of a potential new link between A-to-I editing of B2 SINE RNA and Alzheimer's disease. The information may be interesting to scientists in the RNA editing community, but I think the work is largely observational at this point.

Referees cross-commenting

I agree with reviewer #2 that orthogonal validations will be important to show in the manuscript.

3. How much time do you estimate the authors will need to complete the suggested revisions:

Estimated time to Complete Revisions (Required)

(Decision Recommendation)

Between 1 and 3 months

Review #2

1. Evidence, reproducibility and clarity:

Summary:

Mitchell et al. developed and utilized a novel bioinformatic method to identify specific RNA editing sites in B2 RNA. They then applied this method to published transcriptome-wide data from a mouse model of amyloid beta toxicity and immortalized hippocampal cells treated with amyloid peptides. The main novel finding from this is that amyloid-beta accumulation in hippocampal cells in vivo and in vitro correlates with an increase in RNA editing at site 49 of B2 RNA, which promotes stability of B2 RNA. This data is followed by suggestions that this increased RNA editing is serving as a protective mechanism during the early stages of amyloid accumulation.

While this hypothesis and the novel method to address it is interesting and potentially very useful to the field, in its current form this manuscript remains highly speculative. While there is established evidence to support the authors claims individually, more direct evidence to address the novel RNA editing finding and model is required. Below are comments which would significantly enhance the strength of the novel method and hypothesis.

Major comments:

One major weakness of the paper in its current form is the use of previously published data, with no independent biological cohorts for validation. Instead published immortalized hippocampal cell data is used to validate the published mouse model transcriptome-wide analysis. While it is convenient to use previously published data, and cell culture experiments to test general findings in mice, RNA editing is highly cell-type and state-dependent. For example this statement is supported even within this paper. While the authors observe a variety of sites that are significantly RNA edited in the mouse model, they are only able to replicate site 49 in vitro. This suggests the authors are missing a variety of potentially very biologically important RNA editing sites by only utilizing cell culture experiments.

Therefore, if the authors have remaining cell pellets or purified RNA in the freezer, these validations should be performed directly on these samples. Additionally, generation of a new cohort, while time-consuming and expensive would also allow the authors to directly address their hypothesis and validate their sequencing data. In practice most published work with transcriptome-wide data requires at a minimum an independent cohort to be generated for validation of the findings, and this appears to be absent in this paper.

To this end, the second major weakness of the paper is while the RNA-seq bioinformatic method claims to identify RNA editing at single bases, no specific wet lab validation is performed to support this. This was acceptable when the only method to profile RNA editing was bioinformatics (which the authors have made an improvement upon), but there are now several established methods for validating RNA editing data in cells including: inosine cyanoethylation (ICE-seq https://pubmed.ncbi.nlm.nih.gov/20835228/), and use of Endo V to either cleave (https://www.nature.com/articles/s41593-020-0627-5?proof=t) or enrich (https://currentprotocols.onlinelibrary.wiley.com/doi/abs/10.1002/cpch.82) inosine RNA (e.g. Viper-seq). Even including sanger-sequencing of cDNA products showing a conversion of A-G at these sites from independent samples can be done at a minimum. All of these methods could be performed on the immortalized hippocampal cells fairly cheaply and quickly, or RNA derived from the mouse model which would be far better for addressing the central hypothesis.

In addition to validating the actual RNA editing sites either in vitro or in vivo, the functional outcome of this change within the paper is suggested, but entirely absent experimentally. This is likely due in part to the already published work (used in part for this paper), as well as the other well established data that B2 RNA are known to interact with Pol II established by this laboratory and others. However because the main significant finding from this paper is the RNA editing site at least some effort needs to be put into validating it, and its functional consequences. Specifically, the authors can use the immortalized hippocampal cells with the RNA editing inhibitor, and profile a handful of candidate genes. When doing this the Pol II occupancy and/or altered gene expression can be measured along with the RNA editing levels as measured by above methods. A more sophisticated approach to causally nail this effect would be to utilize dCas13 RNA base editors directed to this site followed by profiling of the effect on Pol II occupancy of target genes and their expression. Either of these data could validate the model presented in Figure 1, as well as the proposed hypothesis.

Without attempt at some of these experiments described above it is the reviewers opinion that this data should not be published in its current form. Even if the authors wish to instead publish this work as a standalone methods paper, at a minimum validation of the method with an orthogonal sample set and method would be required.

Minor comments:

By what mechanism do the authors think that amyloid beta accumulation is inducing increased RNA editing?

Similarly what do the authors think leads to this mechanism being shutdown later in pathology?

I am unclear why you would want to give Adar inhibitors to already impaired animals unless this is to induce an impairment phenotype in wild-type mice? Would you not want to explore enhancing RNA editing early on at a higher level, or reintroducing it when absent in older mice? To this end please see my above comments on using dcas13 RNA base editors to do this site specifically to support the findings.

2. Significance:

Significance (Required)

The bioinformatic method described by the authors, if further validated, could be of use in a variety of tissue and cell types where transcriptome-wide data exists. Additionally, the concept put forward by this paper, and supported by other findings from this and other laboratories, of B2 RNA regulating transcription in response to cellular insults in a variety of contexts is an intriguing mechanism. Again if further validated and expanded in vivo, this concept could then be applied to enhance RNA editing and further enhance this putative counter-measure to toxic effects from amyloid beta accumulation.

This work could either be published as a stand-alone methods paper, or expanded and validated for a conceptual advancement in the field of neuroscience and molecular biology.

The authors area of expertise is cognitive neuroepigenetics including published work on RNA editing and ADAR1 in Nature Neuroscience.

Referees cross-commenting

I believe it is reasonable for all of of reviewer 1's comments to be addressed. Overall the manuscript needs to be refined and expanded more prior to publication.

3. How much time do you estimate the authors will need to complete the suggested revisions:

Estimated time to Complete Revisions (Required)

(Decision Recommendation)

Cannot tell / Not applicable

We are grateful to the reviewers for reviewing our manuscript and for their comments that helped to improve further the quality of the current study. In general, the reviewers agree on the significance and novelty of our bioinformatics approach but have also proposed additional analysis and experiments that "*would significantly enhance the strength of the novel method and hypothesis.*" In particular, they note that "*orthogonal validations will be important to show in the manuscript (Reviewer1)*" and that *"The bioinformatic method described by the authors, if further validated, could be of use in a variety of tissue and cell types where transcriptome-wide data exists (Reviewer 2)".* They also note that *"While there is established evidence to support the authors claims individually, more direct evidence to address the novel RNA editing finding and model is required (Rev.2)"* and that "*While the observations are interesting, the authors can potentially delve deeper into the underlying mechanisms (Rev.1)*". Indeed, the primary aim of the current manuscript has been the development of a novel approach to address the challenges of studying A-to-I editing in RNAs from repeats such as SINEs. Inevitably, by subsequently testing this method in real data from mouse amyloid beta pathology models we came across a novel finding that had eluded until now our attention due to these bioinformatics challenges. Our approach revealed the differential adenosine to inosine RNA editing of SINE B2 RNAs during response to amyloid beta neuro-toxicity in mouse hippocampus but both our method and this finding may have not been extensively validated in our initial submission. We now provide extensive additional validation of both our method and this finding as recommended by the reviewers through new experimental additions. Being a predominantly bioinformatics paper in scope, elucidating the exact mechanism underlying the above finding is beyond the scope of the current manuscript and is tackled in a different manuscript we are currently submitting that focuses on the exact role of editing and *Adar* on B2 RNA processing. In the current revised manuscript, we have instead prioritized the provision of all these orthogonal experiments and independent datasets that could validate our method and primary finding of differential B2 RNA editing during amyloid beta pathology and neuro-toxicity. In particular, we now provide:

<u>Validation of our method through:</u>
- a new set of siRNA experiments targeting *Adar* and *Adarb1* transcripts though siRNAs, and evaluating the efficiency of the knock down and the ability of our bioinformatics method to detect it compared to other approaches.
- application of EndoV treatment assays as an alternative way of confirming differences in editing.

<u>Validation of both our findings and method through:</u>
- an independent sequencing approach (Nanopore direct RNA-seq), that is capable of identifying modified RNA bases, applied to the same samples used to generate our Illumina RNA-seq datasets.

<u>A refinement of our analysis</u> that includes B2 subfamilies and ability to provide better positional specificity of RNA edits.

Validation of our findings through:
- the employment of an independent Illumina RNA-seq dataset and another mouse model produced from another lab.

To this end, the revised version of our manuscript now includes:
- Three new main figures (Fig.2, Fig.4 and Fig.6) and multiple revised figure panels in two of the other Figures (Fig. 3, and Fig.5),
- Eight new supplementary figures with multiple panels each (Supplementary Figures 1-8),

We have also revised the main text accordingly.

Please find below the reviewers 'comments and a detailed point by point response to these comments. A document with the changes from the initially submitted manuscript being highlighted is attached at the end of this response.

Point-by-point description of the revisions

REVIEWER 1

Reviewer #1 (Evidence, reproducibility and clarity (Required)):

This reviewer generally remarks that: "*In the submitted manuscript, the authors explored ADAR-mediated A-to-I editing in B2 sine RNAs. They developed a computational approach to measure editing rates at a population level and found that editing at certain positions in the B2 RNA was dysregulated in mouse hippocampal cells suffering from amyloid beta toxicity. While the observations are interesting, the authors can potentially delve deeper into the underlying mechanisms. Below are some comments that I have for improving the manuscript.*"

We appreciate the encouraging comments made by this reviewer.

Major comments:

Point 1. The reviewer notes that "*After mapping the reads to the REPome, the authors calculated the proportions of base compositions within the whole population of B2 RNAs instead of calculating the RNA editing rate for each distinct member of the REPome. What advantage(s) are there to the authors' reported method vis-a-vis a more conventional approach of simply calculating the editing levels for each distinct B2 RNA "gene" (i.e. member of the REPome)?*

We thank the reviewer for this insightful comment. In response, we have clarified the advantages of our population-based approach in the revised manuscript (page 8, lines 19–31) and added new supporting analyses (Supplementary Figure 2A–C). As shown in Supplementary Figure 2A, REPome-based alignment captures a substantially larger number of informative reads than genome-based mapping, thereby reducing the loss of multi-mapping reads that is intrinsic to conventional approaches. Supplementary Figure 2B presents A-to-I editing estimates generated by our method, which quantifies relative editing across the entire expressed population of B2 RNAs. By contrast, Supplementary Figure 2C shows results obtained using a conventional strategy that calculates editing levels independently for each distinct B2 RNA sequence and subsequently aggregates these values across positions and samples. Our approach robustly detects the expected reduction in RNA editing in AntiAdar samples. In these samples, both *Adar* and *Adarb1* expression and activity are reduced (Figure 2E,F; Supplementary Figure 1), and correspondingly lower B2 RNA editing is clearly observed relative to Scramble controls (Figure 2B–D; Supplementary Figure 2B). This difference is not captured by the conventional method (Supplementary Figure 2C), which yields nearly uniform editing levels along the entire length of B2 RNAs, lacking any positional specificity. Such homogeneous distributions—where adjacent positions exhibit almost identical editing rates—are inconsistent with described editing preferences by ADAR enzymes. In contrast, our population-based method preserves this positional specificity, revealing distinct editing "hotspots" that align with and often cluster around known ADAR substrate structures (Figure 2C,3C, 5C and new text at page 8, lines 13-18).

Together, these analyses demonstrate that our population-based REPome alignment and aggregate editing rate calculation retain biological sensitivity and positional specificity, improving accuracy in quantifying A-to-I editing within repetitive elements such as B2 RNAs.

Point 2. The reviewer asks: *"Figure 2C: What is the p-value (WT vs APP) for 3m, 6m, and 12m?"*

Note: The equivalent of the previous Figure 2C is now presented as Figure 3B in the revised manuscript.

We have now included values derived from Kolmogorov–Smirnov (KS) tests to quantify the differences in cumulative A-to-I editing distributions between APP and WT mice at each age group (3m, 6m, and 12m). These KS values are now reported in the revised figure, its legend and the text (Figure 3B) as well as in similar figures across the manuscript (Figure 2B and 5B, and Supplementary Figure 4B).

Additionally, to improve positional interpretation, we have modified the plots so that the x-axis in Figures 3 B and C reflects the ordinal nucleotide position along the B2 mm1a consensus sequence rather than a rank-ordered representation. This change, which has been also applied to other figures (Figures 2 B and C, 5 B and C and Supplementary Figure 4 B and C) allows direct comparison of positional editing distributions between sample groups.

Statistical values per editing position are also now provided for all the above comparisons (Supplementary Figures 3, 5 and 6).

Point 3. The reviewer asks: *"Figure 4: Is the editing of position 49 increased in all members of the REPome or is the increase observed only in certain B2 RNAs? The location of the adenosine at position 49 (figure 3A) also appears to be non-ideal for ADAR-mediated editing. Are there any sequence variations in*

the B2 RNA that can produce a more stem-like structure around position 49? Perhaps these are the SINE RNAs whose editing is increased upon treatment with b-amyloid peptide."

Note: Old figure 4 corresponds now to new Figure 5 and associated Supplementary Figure 6.

We thank the reviewer for this valuable comment. In response to this, firstly, as mentioned above in point 2, we have updated all plots to reflect positional distribution of the editing rates (instead of rank-order position) so that they allow a better comparison with regional B2 RNA structures (see new Figures 2C, 3C and 5C that show the exact position of editing peaks). As also shown in our revised methods section, we have been able to refine our edit-rate calculation by making changes in the part of our algorithm that performs normalization in order to reduce bias at low-coverage or compositionally extreme positions (page 18, lines 4-26). To this end we have been able to generate and now provide a complete list of all differentially edited positions. Beyond position 49 this list includes seven other positions all of which are common both *in vivo* (APP vs WT, Figure 3 and Associated Supplementary figure 3, Supplementary Figure 4 and associated Supplementary Figure 5) and *in vitro* (HT22 42 vs R, Figure 5 and associated Supplementary Figure 6).

In addition, to address whether changes in B2 RNA sequences affect our results, we have now added a new supplementary figure that shows that the increase in A-to-I editing is confined to the B2 mm1a and B2 Mm1t subfamilies, and not to the third B2 subfamily (Mm2 elements) (Supplementary Figure 8 and new text at page 14 lines 1-9). This analysis indicates that indeed the editing signal arises mainly from certain B2 RNA subfamilies rather than being uniformly distributed across all of them. For this reason, to improve normalization and cross-sample comparability, our refined analysis in the revised manuscript focused on the mm1a subfamily which is also the subfamily most closely aligned to the previously published B2 consensus sequence and the one studied with regards to B2 RNA ribozyme activity and RNA Polymerase II suppression in the past. Our new analysis also includes only adenosine-containing positions from that consensus sequence**.** As mentioned above, this refinement enhanced positional resolution and revealed additional editing sites beyond position 49 in both hippocampal tissue and HT-22 cells. Interestingly, these sites exhibit reproducible editing patterns across our datasets, in positions that are consistent with some internal double-stranded RNA structures described for B2 RNAs (Espinoza et al., 2007; Podbevsek et al., 2018; Sharma et al., 2024). However, it needs to be noted that formation of double stranded structures in other regions through for example inverted B2 repeats as in case of Alu RNAs in human is also very likely, so the alignment between editing patterns and the structure that produced them requires much more further analysis. This is now discussed in the text at page 13 lines 17-24.

Point 4. The reviewer asks: *"Figure 5A-B: What are the p-values?"*

As noted above, the relationship between B2 RNA processing ratio and editing rate is no longer included in this paper and will be explored thoroughly in an upcoming paper.

Point 5. The reviewer remarks: "*Figure 5C-F: (a) How do we know that 8-AzaA treatment was effective in blocking the activity of ADAR1? Can the authors check the editing levels of some known ADAR1*

substrates in the control and treated cells? (b) 8-AzaA may have off-target effects in HT-22 cells. The authors should use an orthogonal method (e.g. siRNA knockdown) to confirm the results."

We thank the reviewer for this comment. Indeed, previous studies have reported that 8-AzaA may exhibit off-target and pleiotropic effects—including inhibition of general transcriptional and nucleotide metabolic processes—rather than selective ADAR1 inhibition (for example Cottrell et al, Cancer Res Commun 2021). Thus, we have now replaced the 8-AzaA analysis with an siRNA KD experiment dataset in the revised manuscript (new Figure 2, Supplementary figure 1), which provides a clearer and mechanistically more reliable validation of ADAR-dependent editing effects. In particular, in line with the Reviewer's suggestion, we conducted siRNA-mediated knockdown of *Adar* and *Adarb1*, which resulted in a clear reduction in transcript levels (Figure 2E) and a corresponding decrease in editing activity, as demonstrated by our EndoV assays (new Figure 2F). This experiment provided a more specific and reproducible means of validating ADAR-dependent editing than 8-AzaA.

Point 6. The reviewer asks: *" What happens when HT-22 cells are treated with both b-amyloid peptide and 8-AzaA? Will we see an even more pronounced amount of B2 RNA processing?" and ""How does A-to-I editing confer a protective effect? Does editing prevent EZH2 from interacting with B2 RNA""*

We thank the reviewer for this insightful follow-up questions. As mentioned above, in the revised version of the manuscript, we have discontinued the use of 8-Azaadenosine (8-AzaA) because of its now better-documented lack of specificity and off-target effects, as discussed above. Instead, we now employ siRNA-mediated knockdown of *Adar* and *Adarb1* in these cells, which provides a more direct and specific means of modulating A-to-I editing activity. Our new data demonstrates that Adar / Adarb1knockdown alone significantly reduces A-to-I editing and correlates with decreased expression of B2 stress-response genes (Figure 6C), consistent with a regulatory role of ADAR activity in B2 RNA–mediated transcriptional control. However, the mechanistic interplay between ADAR modulation, A to I editing and B2 RNA processing will be comprehensively presented in a separate manuscript that specifically focuses on how Adar dependent editing influences B2 RNA stability and processing dynamics.

Minor comments:

1. In the introduction, the authors wrote that "multiple recent studies have revealed important functions for SINE genomic elements ... cryptic splicing sites and RNA circularization". No citations have been provided for the post-transcriptional functions (splicing and circularization). Please cite accordingly.

These new references are now provided. (Lin et al; Jeck et al)

2. Typo on page 9: the authors wrote "A-to-I editing levels were found to correlate positively (r = 0.45) with full length B2 RNA levels (Fig.4A)". I think it should be Fig. 5A.

Figures connected with B2 RNA stability are not presented anymore as noted above.

Reviewer #1 (Significance (Required)):

The authors have provided some evidence of a potential new link between A-to-I editing of B2 SINE RNA and Alzheimer's disease. The information may be interesting to scientists in the RNA editing community, but I think the work is largely observational at this point.

Referees cross-commenting

I agree with reviewer #2 that orthogonal validations will be important to show in the manuscript.

REVIEWER 2

Reviewer #2 (Evidence, reproducibility and clarity (Required)):

Summary:

Mitchell et al. developed and utilized a novel bioinformatic method to identify specific RNA editing sites in B2 RNA. They then applied this method to published transcriptome-wide data from a mouse model of amyloid beta toxicity and immortalized hippocampal cells treated with amyloid peptides. The main novel finding from this is that amyloid-beta accumulation in hippocampal cells in vivo and in vitro correlates with an increase in RNA editing at site 49 of B2 RNA, which promotes stability of B2 RNA. This data is followed by suggestions that this increased RNA editing is serving as a protective mechanism during the early stages of amyloid accumulation.

This reviewer generally remarks that "*While this hypothesis and the novel method to address it is interesting and potentially very useful to the field, in its current form this manuscript remains highly speculative. While there is established evidence to support the authors claims individually, more direct evidence to address the novel RNA editing finding and model is required. Below are comments which would significantly enhance the strength of the novel method and hypothesis.*"

We appreciate the encouraging comments made by this reviewer.

Major comments:

<u>Point 1.</u> The reviewer remarks that: "*One major weakness of the paper in its current form is the use of previously published data, with no independent biological cohorts for validation. Instead published immortalized hippocampal cell data is used to validate the published mouse model transcriptome-wide analysis. While it is convenient to use previously published data, and cell culture experiments to test general findings in mice, RNA editing is highly cell-type and state-dependent. For example this statement is*

supported even within this paper. While the authors observe a variety of sites that are significantly RNA edited in the mouse model, they are only able to replicate site 49 in vitro. This suggests the authors are missing a variety of potentially very biologically important RNA editing sites by only utilizing cell culture experiments.

Therefore, if the authors have remaining cell pellets or purified RNA in the freezer, these validations should be performed directly on these samples. Additionally, generation of a new cohort, while time-consuming and expensive would also allow the authors to directly address their hypothesis and validate their sequencing data. In practice most published work with transcriptome-wide data requires at a minimum an independent cohort to be generated for validation of the findings, and this appears to be absent in this paper."

We thank the reviewer for this important comment and fully agree that validation in independent and different biological cohorts and tissues strengthens the robustness of transcriptome-wide analyses. To address this, firstly we have now included an additional dataset derived from a publicly available mouse model of amyloid beta pathology (GEO accession GSE136861), which provides whole-brain RNA-seq data. The results of this independent validation are presented in Supplementary Figure 4 and associated Supplementary Figure 5. This dataset provides the opportunity of testing our approach in an amyloid pathology mouse model dataset produced from another lab from brain tissue. Analysis of this dataset recapitulates the same trend observed in our original model, with A-to-I editing levels high in amyloid beta pathology mouse animals of similar age (Supplementary Figure 4 and Supplementary Figure 5), consistent with our main findings.

In addition, as we did have RNA left from the samples our eLife paper, from which the Illumina RNA-seq data used in Figure 3 originate, we have employed a completely independent RNA sequencing method, Nanopore direct RNA-seq, that has the potential of identifying non standard RNA bases. Using direct RNA-seq we tested both APP mice and HT22 cells from our eLIFE paper cohort and as a control the new AntiAdar siRNA treated HT22 samples RNA of Figure 2. In both cases, direct RNAseq confirmed in these positions the existence of a non-standard adenosine in levels higher in APP mice and amyloid beta peptide treated HT22 cells as well as in the scramble HT 22 cells, consistent with our Illumina RNA-seq findings (Figure 4 and new results section pages 10-11).

As mentioned also in response to Reviewer 1 point #3 and our revised methods section, we have been able to refine our editing-rate calculation in order to reduce bias at low-coverage and compositionally extreme positions. To further enhance biological specificity, we performed our analysis to reads of the B2 Mm1a subfamily that align more closely to the B2 consensus sequence, and performed statistical tests and comparisons of editing rates at positions corresponding to adenosines. Such position-level analyses of these sites, now provided in Supplementary Figures 3, 5, and 6, offer detailed views of editing changes per position across conditions and reveal consistency across RNAs from mouse models from our illumina cohort, the external mouse illumina RNA-seq cohort (Supplementary Figure 4 and 5), the Nanopore direct RNA-seq data (Figure 4C), the HT-22 amyloid beta treated samples (Figure 5 and Supplementary Figure 6) and the HT22 Adar/Adarb1 siRNA experiment (Figure 2).

Together, these updates strengthen the generalizability and reproducibility of our findings by incorporating an independent dataset, an independent method, improving normalization, and refining subfamily- and position-specific analyses.

Point 2. The reviewer notes that "*To this end, the second major weakness of the paper is while the RNA-seq bioinformatic method claims to identify RNA editing at single bases, no specific wet lab validation is performed to support this. This was acceptable when the only method to profile RNA editing was bioinformatics (which the authors have made an improvement upon), but there are now several established methods for validating RNA editing data in cells including: inosine cyanoethylation (ICE-seq https://pubmed.ncbi.nlm.nih.gov/20835228/), and use of Endo V to either cleave (https://www.nature.com/articles/s41593-020-0627-5?proof=t) or enrich (https://currentprotocols.onlinelibrary.wiley.com/doi/abs/10.1002/cpch.82) inosine RNA (e.g. Viper-seq). Even including sanger-sequencing of cDNA products showing a conversion of A-G at these sites from independent samples can be done at a minimum. All of these methods could be performed on the immortalized hippocampal cells fairly cheaply and quickly, or RNA derived from the mouse model which would be far better for addressing the central hypothesis.*"

We thank the reviewer for the suggestion. We have utilized EndoV treatment to demonstrate lowered editing rate in our siRNA knockdown of Adar1 and Adar2 samples (please see new Figure 2F). As noted in the previous point we have also employed Nanopore directRNA-seq for a single base resolution of non-standard adenosines in RNAs from both our mouse model and the AntiAdar treated HT22 cells.

Point 3. The reviewer remarks that "*In addition to validating the actual RNA editing sites either in vitro or in vivo, the functional outcome of this change within the paper is suggested, but entirely absent experimentally. This is likely due in part to the already published work (used in part for this paper), as well as the other well established data that B2 RNA are known to interact with Pol II established by this laboratory and others. However because the main significant finding from this paper is the RNA editing site at least some effort needs to be put into validating it, and its functional consequences. Specifically, the authors can use the immortalized hippocampal cells with the RNA editing inhibitor, and profile a handful of candidate genes. When doing this the Pol II occupancy and/or altered gene expression can be measured along with the RNA editing levels as measured by above methods. A more sophisticated approach to causally nail this effect would be to utilize dCas13 RNA base editors directed to this site followed by profiling of the effect on Pol II occupancy of target genes and their expression. Either of these data could validate the model presented in Figure 1, as well as the proposed hypothesis.*

We now provide gene expression levels and connection with editing levels of both Adar genes and B2 RNA target genes (B2 SRGs) in both mouse and amyloid beta peptide treated HT22 cells as well as cells treated with the AntiAdar siRNAs (New Figure 6, new Suppl. Figure 7). These results have spurred further our interest in investigating more deeply the exact mechanisms underlying B2 RNA stability, Adar activity and editing in a separate paper. If necessary we would be happy to share these results with the reviewers but we

would prefer to not provide them here as this response to reviewers may also be published in advance of that paper.

Without attempt at some of these experiments described above it is the reviewers opinion that this data should not be published in its current form. Even if the authors wish to instead publish this work as a standalone methods paper, at a minimum validation of the method with an orthogonal sample set and method would be required."

Minor comments:

By what mechanism do the authors think that amyloid beta accumulation is inducing increased RNA editing?

As shown in new Figure 6 an increase in the *Adar* (and *Adarb1*) levels during response to amyloid beta toxicity is a major cause behind the increase in editing (see also confirmation of the impact of knocking down *Adar* and *Adarb1* on B2 RNA editing in new Figures 2 and Supplementary Figure 1).

Similarly, what do the authors think leads to this mechanism being shutdown later in pathology?

This is an excellent_question, and_based on data in_new Fig 7, Adar enzymes levels contribute to this. However, the exact upstream mechanisms remain elusive.

I am unclear why you would want to give Adar inhibitors to already impaired animals unless this is to induce an impairment phenotype in wild-type mice? Would you not want to explore enhancing RNA editing early on at a higher level, or reintroducing it when absent in older mice? To this end please see my above comments on using dcas13 RNA base editors to do this site specifically to support the findings.

We apologize for the confusion created here. In our initial discussion session we should have proposed the use of Adar inhibitors in mice as a way to confirm *in vivo* a correlation between editing, B2 RNA stability and progression of neurodegeneration. This part has now been revised (last paragraph in Discussion, page 15).

Reviewer #2 (Significance (Required)):

The bioinformatic method described by the authors, if further validated, could be of use in a variety of tissue and cell types where transcriptome-wide data exists. Additionally, the concept put forward by this paper, and supported by other findings from this and other laboratories, of B2 RNA regulating transcription in response to cellular insults in a variety of contexts is an intriguing mechanism. Again if further validated and expanded in vivo, this concept could then be applied to enhance RNA editing and further enhance this putative counter-measure to toxic effects from amyloid beta accumulation.

This work could either be published as a stand-alone methods paper, or expanded and validated for a conceptual advancement in the field of neuroscience and molecular biology.

The authors area of expertise is cognitive neuroepigenetics including published work on RNA editing and ADAR1 in Nature Neuroscience.

Referees cross-commenting

I believe it is reasonable for all of of reviewer 1's comments to be addressed. Overall the manuscript needs to be refined and expanded more prior to publication.

April 2, 2026

RE: Life Science Alliance Manuscript #LSA-2026-03668-T

Dr. Athanasios Zovoilis
University of Manitoba
Biochemistry and Medical Genetics
Canada

Dear Dr. Zovoilis,

Thank you for submitting your revised manuscript entitled "Differential A to I editing of B2 RNAs unveils a novel type of response to amyloid β neuro-toxicity". We returned this to the original reviewers from Review Commons and their comments are below. As you will see both reviewers commented positively on the revised work while making suggestions for further improvement. We invite you to consider requests from both reviewers to improve the clarity of the results and refine the discussion. Including the additional data requested by Reviewer 1 is left to your discretion. We would be happy to publish your paper in Life Science Alliance pending these changes as well as final revisions necessary to meet our formatting guidelines.

MANUSCRIPT ORGANIZATION AND FORMATTING:

To avoid unnecessary delays in the acceptance and publication of your paper, please read the following information carefully. Full guidelines are available on our Instructions for Authors page, https://www.life-science-alliance.org/authors

Submission of a paper that does not conform to Life Science Alliance guidelines will delay the acceptance of your manuscript.

-Please upload your main manuscript text as an editable doc file.
-Please upload your main and supplementary figures as single files.
-Please add a Running Title in our system.
-Please add ORCID ID for corresponding author - you should have received instructions on how to do so.
-Please add a Summary Blurb/Alternate Abstract and a Category in our system.
-Please add the X and Bluesky handles of your host institute/organization, as well as your own, and/or one of the authors, in our system.
-The titles in both the system and the manuscript file must be consistent with each other.
-Please enter the author's contributions in our system, as well.
-Please use the [10 author names et al.] format in your references (i.e., limit the author names to the first 10).
-Please add your main and supplementary figure legends to the main manuscript text after the references section.
-Please add a "Data Availability" section after the Materials & Methods section. This section should include the Illumina and Nanopore datasets generated in this study. The current section describing sequencing datasets generated from prior studies can remain. Please consult our guidelines at https://www.life-science-alliance.org/manuscript-prep#format
-Please add callouts for Figures S1A-B; S2A-F; S3A-B; S5A-B; S6A-B and S8A-C to your main manuscript text.

We encourage our authors to provide original source data, particularly uncropped/-processed electrophoretic blots and spreadsheets for the main figures of the manuscript. If you would like to add source data, we would welcome one PDF/Excel-file per figure for this information. These files will be linked as supplementary "Source Data" files.

It is Life Science Alliance policy that if requested, original data images must be made available to the editors. Failure to provide original images upon request will result in unavoidable delays in publication. Please ensure that you have access to all original data images prior to final submission.

If you are planning a press release on your work, please inform us immediately to allow informing our production team and scheduling a release date.

We welcome submissions of potential cover images for the issue of LSA in which your work would appear. If you have high quality images associated with this work, please feel free to email these, with a caption, to the journal office.

LSA encourages authors to provide a 30-60 second video where the study is briefly explained. We will use these videos on social media to promote the published paper and the presenting author (for examples, see https://docs.google.com/document/d/1-UWCfbE4pGcDdcgzcmiuJl2XMBJnxKYeqRvLLrLSo8s/edit?usp=sharing). Corresponding or first-authors are welcome to submit the video. Please submit only one video per manuscript. The video can be emailed to contact@life-science-alliance.org

FINAL FILES:

To upload the final version of your manuscript, please log in to your account: https://lsa.msubmit.net/cgi-bin/main.plex
You will be guided to complete the submission of your revised manuscript and to fill in all necessary information. Please get in touch in case you do not know or remember your login name.

The following items are required for acceptance.

-- An editable version of the final text (.DOC or .DOCX) is needed for copyediting (no PDFs).

-- High-resolution figure, supplementary figure and video files uploaded as individual files: See our detailed guidelines for preparing your production-ready images, https://www.life-science-alliance.org/authors

-- Summary blurb (enter in submission system): A short text summarizing in a single sentence the study (max. 200 characters including spaces). This text is used in conjunction with the titles of papers, hence should be informative and complementary to the title. It should describe the context and significance of the findings for a general readership; it should be written in the present tense and refer to the work in the third person. Author names should not be mentioned.

The license to publish form must be signed before your manuscript can be sent to production. A link to the license to publish form will be available to the corresponding author only. Please take a moment to check your funder requirements.

Reviews, decision letters, and point-by-point responses associated with peer-review at Life Science Alliance will be published, alongside the manuscript. If you do want to opt out of having the reviewer reports and your point-by-point responses displayed, please let us know immediately.

Thank you for your attention to these final processing requirements. Please revise and format the manuscript and upload materials as soon as you are able.

Thank you for this interesting contribution to the literature. We look forward to publishing your paper in Life Science Alliance.

Sincerely,

Tim Fessenden
Scientific Editor
Life Science Alliance

Reviewer #1 (Comments to the Authors (Required)):

In the submitted manuscript, the authors presented their study of RNA editing in mouse B2 SINE RNAs. Since they have focused on Alzheimer's disease, the topic is of clinical relevance and their results on editing changes in the disease state might be of interest to the scientific community. Below are some suggestions that might improve their work further.

Major comments:

1) Besides qPCR (Figure 2E), there should also be a Western blot showing substantial decrease of ADAR1/ ADAR2 protein levels with siRNA treatment.

2) Figure 2 should include a panel showing the relevant dsRNA structures marked with the corresponding editing hotspots. Is there anything special about the editing hotspots?

3) Instead of combined knockdown ("AntiADAR"), it would be useful to examine ADAR1 and ADAR2 individually. The authors wrote that "both enzymes demonstrably can edit B2 RNAs, though with potentially different efficiencies". Which sites are ADAR1 targets and which are ADAR2 targets? Moreover, Alu repeats in human are predominantly ADAR1 targets, so it's interesting that ADAR2 appears to play a bigger role in the mouse.

4) Instead of xPORE, the authors should use deep learning models that can detect A-to-I editing in native RNA directly.

5) Figure 3B: It seems like A-to-I editing starts off being higher in APP mice and then decreases over lifespan and drops to a level lower than that in wildtype controls by 12 months. Are there any clues in the gene expression data that might provide an explanation to this pattern?

Minor comment:

6) Figure 2E should appear earlier. Editing analysis should follow only after confirmation of successful knockdown.

Reviewer #2 (Comments to the Authors (Required)):

Peer Review
1. Summary
This manuscript by Mitchell, Saville, and colleagues presents two interconnected contributions: a novel repeat-aware bioinformatics framework for quantifying position-specific adenosine-to-inosine (A-to-I) RNA editing within SINE B2 non-coding RNAs, and the application of this framework to reveal a previously unrecognized epitranscriptomic response to amyloid beta neurotoxicity.
The methodological advance centers on a "REPome"-based approach that circumvents the well-known problems of multi-mapping reads and sequence heterogeneity that plague standard genome-aligned editing pipelines when applied to repetitive elements. By collapsing identical B2 sequences into unique representatives and treating the expressed population as a collective, the authors achieve population-level, position-specific editing quantification that standard methods cannot reliably deliver.
Applying this framework, the authors identify discrete A-to-I editing hotspots within B2 mm1a RNAs - concentrated in regions predicted to form double-stranded RNA structures - whose editing levels are selectively elevated during the earliest stages of amyloid beta pathology. This finding is reproduced across two independent mouse models (APPNL-G-F and APPB6.APP/PS1), in hippocampal cell culture exposed to acute amyloid beta peptide toxicity, and validated using an orthogonal sequencing technology (Oxford Nanopore direct RNA-seq). Functional perturbation via siRNA-mediated knockdown of Adar/Adarb1 confirms ADAR enzyme dependence of the identified editing events and demonstrates that reducing ADAR activity suppresses expression of B2 RNA-regulated stress-response genes (B2 SRGs). Together, the work defines a regulatory axis linking amyloid beta toxicity → ADAR upregulation → site-specific B2 RNA editing → B2 SRG expression, with potential relevance to conserved mechanisms in Alzheimer's disease.
This is a significantly improved manuscript relative to its initial submission. The authors have responded substantively to prior reviewer concerns by replacing the non-specific 8-AzaA inhibitor with siRNA knockdown experiments, adding EndoV biochemical validation, incorporating Nanopore direct RNA-seq as an orthogonal sequencing approach, including an independent external mouse dataset, and refining the bioinformatics pipeline to improve positional resolution at the subfamily level. These additions substantially strengthen both the methodological and biological claims.

---

2. Assessment of Main Points
Point 1: The REPome-based pipeline outperforms standard genome-aligned approaches for B2 RNA editing quantification
Assessment: Well supported.
The comparison presented in Supplementary Figure 2 is informative and directly addresses the prior reviewer concern. The authors demonstrate that genome-based alignment captures substantially fewer reads mapping to B2 elements (Suppl. Fig. 2A), and that the conventional edit-then-aggregate approach produces an artifactually flat, positionally uninformative editing distribution that fails to detect the expected ADAR-dependent signal (Suppl. Fig. 2C). By contrast, the REPome-based, group-then-edit-rate approach faithfully recovers the reduction in editing caused by Adar/Adarb1 knockdown, with clear positional hotspots consistent with known dsRNA-forming regions of B2 RNA (Suppl. Fig. 2B, Fig. 2B-D). The logic underpinning the population-level proportion approach is sound: by treating variability among B2 copies as a distributional property rather than a mapping problem, the method avoids both read loss and locus misassignment. No additional experiments are required to support this claim.
One clarification that would benefit readers: the Methods section describes filtering for mm1a elements specifically after initially constructing the REPome from mm1a, mm1t, and mm2 sequences. It would be helpful to explicitly state in the Results or Methods whether reads that align better to mm1t or mm2 elements are excluded prior to the position-specific analysis, and how cross-subfamily alignment ambiguity is handled. This does not require new experiments - a sentence or two of clarification suffices.
Point 2: A-to-I editing is increased at specific B2 RNA positions during early amyloid beta pathology in the mouse hippocampus
Assessment: Well supported, with minor caveats regarding statistical power at the 3-month timepoint.
The cumulative and position-specific editing analyses in APP versus WT mice at 3, 6, and 12 months (Fig. 3B-D) present a coherent picture: editing is elevated early (3m and 6m) and this increase reverses at the terminal stage (12m), consistent with a dynamic epitranscriptomic response rather than a static feature. The eight positions identified as significantly differentially edited (Suppl. Fig. 3) are reproducible across datasets, which is an important strength.
One limitation worth acknowledging more explicitly in the text is that the 3-month WT group contains only n=2 animals, which limits statistical power for that comparison. The authors note this in the figure legend but the Discussion does not explicitly caveat the 3-month findings accordingly. Given that the pre-symptomatic timepoint is arguably the most interesting from a biomarker standpoint, a brief acknowledgment of this limitation in the Discussion would be appropriate. This is a text change only.
Point 3: Findings replicate in an independent external dataset (APPB6.APP/PS1 model)

Assessment: Well supported.
The replication of the elevated editing pattern in whole-brain tissue from an independent mouse model generated by a different laboratory (GEO: GSE136861), presented in Supplementary Figures 4 and 5, is a meaningful addition. The overlap of significantly edited positions between the two models (Suppl. Fig. 4D) and the parallel direction of editing changes at the eight-position subset (Suppl. Fig. 5) substantially mitigate concerns about model-specificity or laboratory-specific batch effects. No additional experiments are required.
It would be worth noting explicitly that this external dataset uses whole-brain tissue rather than hippocampus-specific dissection, and that the transgene approach (human APP and PSEN1 overexpression) differs genetically from the APP knock-in strategy. The authors are aware of these differences but they are only briefly mentioned. Acknowledging these distinctions more directly in the Results - and noting them as a strength of cross-model replication rather than a confound - would help readers contextualize the generalizability of the findings.
Point 4: Nanopore direct RNA-seq independently supports elevated adenosine modification at B2 RNA positions
Assessment: Supportive, with an important and appropriately acknowledged caveat regarding modification specificity.
The Nanopore data (Fig. 4) provide a valuable orthogonal line of evidence. Both the Xpore-computed modification rate distributions and the raw Nanopolish signal density plots at the eight identified positions show directionally consistent patterns: higher modification signals in Scramble versus AntiAdar cells, and in 6-month APP versus WT hippocampi. The KS statistics (KS=0.001 and KS=0.002, respectively) indicate meaningful distributional differences.
The authors appropriately note in the Discussion that the R9.4.1 flowcell chemistry cannot unambiguously distinguish inosine from other RNA modifications, and that other base modifications or ADAR-independent crosstalk cannot be excluded. This caveat is honest and scientifically appropriate. Position 112 showing no difference in Nanopore signal despite being identified as significantly edited by Illumina sequencing is also acknowledged - the authors should briefly speculate in the Discussion whether this reflects lower coverage at that position, a local sequence context unfavorable for nanopore signal discrimination, or a potential false positive in the Illumina-based analysis. No additional experiments are needed; this is a minor interpretive addition.
Point 5: ADAR knockdown validates pipeline sensitivity and links editing to B2 SRG expression
Assessment: Well supported.
The siRNA knockdown of Adar and Adarb1 (Fig. 2) is a significant improvement over the previously used 8-AzaA inhibitor. Knockdown efficiency is confirmed at both the transcript level (Fig. 2E, RT-qPCR; Suppl. Fig. 1, RNA-seq) and functionally via the EndoV inosine-cleavage assay (Fig. 2F). The EndoV result is particularly valuable as it provides a sequence-independent biochemical confirmation that inosine-containing B2 RNA transcripts are depleted following ADAR knockdown. The correlation between Adar/Adarb1 expression and cumulative editing rate across individual replicates (Suppl. Fig. 1, R=0.78 and R=0.91) is compelling.
The connection between ADAR activity and B2 SRG expression (Fig. 6C) is logically presented and the directionality is consistent: knockdown reduces editing and correspondingly reduces B2 SRG expression. The per-replicate correlations between cumulative editing and B2 SRG expression (Suppl. Fig. 7) further support this coupling. The authors are appropriately cautious about mechanistic interpretation, correctly noting that the exact causal pathway linking editing to SRG expression will be addressed in a forthcoming manuscript. No additional experiments are required for the claims made in this paper.
Point 6: Acute amyloid beta peptide exposure recapitulates the in vivo editing pattern in HT-22 cells
Assessment: Well supported.
The in vitro model (Fig. 5) shows that 6-hour exposure to amyloid beta 1-42 peptides increases B2 RNA editing at the same positional hotspots identified in APP mice, with directionally consistent changes at the eight-position subset (Suppl. Fig. 6). The consistency across in vivo and in vitro systems - and across the acute toxicity model and the chronic pathology model - is a meaningful strength of the dataset as a whole.

---

3. Additional Issues
Text and presentation:
• The abstract refers to "hippocampal or neocortical tissue from two independent mouse models." The neocortex mention appears to refer to the whole-brain external dataset. The authors should clarify in the abstract and Results that the external validation dataset uses whole-brain tissue (which includes but is not restricted to neocortex), to avoid overstating regional specificity.
• The Discussion (page 13) states this is "the first study in hippocampus to identify specific positions within SINE RNAs that act as hotspots of A-to-I editing." Given that the external validation dataset uses whole-brain tissue and the in vitro work uses a hippocampal-derived cell line, the phrasing should perhaps be broadened slightly, or more carefully qualified to distinguish hippocampal tissue findings from cell line findings.
• Figure 4C legend refers to "position 70" as showing identical distributions corresponding to non-detectable modification changes. However, position 70 does not appear in the list of the eight significantly edited positions in Figure 3D, which is consistent. It would be helpful to add a brief parenthetical noting that position 70 serves as an internal negative control in this panel, to assist readers in interpreting the layout.
• The Methods section describes REDItools parameters including setting minimum editing frequency to 0 for "maximum sensitivity." While the rationale for other parameters is explained, the authors might briefly note that setting -n 0 could in principle introduce false positives at positions with very low coverage, and explain how the subsequent population-based approach and the editing rate threshold of $>10^{-4}$ mitigate this concern.
Statistics:
• Supplementary Figures 3B, 5B, and 6B present one-tailed Student's t-tests. The choice of one-tailed testing is presumably justified by the directional hypothesis (editing is increased in disease/amyloid conditions). This should be stated explicitly in the

figure legends or a consolidated statistical methods section, as one-tailed tests are sometimes viewed critically without clear a priori justification.

• The authors report KS test values for cumulative distribution comparisons throughout but do not specify whether these are two-sample KS tests. This should be clarified in the Methods.

Minor:

• Page 7, Results: "Adar1b1" appears to be a typographical error for "Adarb1."

• The GEO accession number for the new Illumina and Nanopore data (GSE316378) is provided in the Methods, which is appreciated. Authors should confirm this accession is publicly accessible at the time of publication.

• Supplementary Figure 8 comparing editing across mm1a, mm1t, and mm2 subfamilies is informative and the observation that mm2 displays divergent editing behavior is interesting. The authors might consider adding one sentence in the Discussion noting whether the absence of elevated editing in mm2 elements - which lack the conserved ribozyme motifs - could be consistent with a model linking editing sites to functional RNA secondary structures. This is a minor interpretive point that does not require new data.

Final Recommendation

I am pleased to endorse this manuscript for publication in its current state. The authors have produced a rigorous, well-validated study that makes a genuine methodological contribution to the field of RNA editing analysis and uncovers a biologically compelling epitranscriptomic response to amyloid beta neurotoxicity. The revisions undertaken in response to prior review have been thorough and have meaningfully strengthened the manuscript. The points raised above are minor in nature and can be addressed at the discretion of the authors and editors without further review. I commend the authors for the quality and scope of this work.

Manuscript number: LSA-2026-03668-T

Corresponding author(s): Athanasios, Zovoilis

We are grateful to the reviewers for reviewing the manuscript and for their comments that helped to improve further the quality of the current work. Please find below the reviewers 'comments and a detailed point by point response to these comments. A document with the changes from the initially submitted manuscript being highlighted is attached at the end of this response.

Point-by-point response to the Reviewers' comments.

REVIEWER 1

Reviewer #1 (Comments to the Authors (Required)):

This reviewer generally remarks that: "*In the submitted manuscript, the authors presented their study of RNA editing in mouse B2 SINE RNAs. Since they have focused on Alzheimer's disease, the topic is of clinical relevance and their results on editing changes in the disease state might be of interest to the scientific community. Below are some suggestions that might improve their work further.*"

Major comments:

Point 1. The reviewer notes that *"Besides qPCR (Figure 2E), there should also be a Western blot showing substantial decrease of ADAR1/ ADAR2 protein levels with siRNA treatment.*

We thank the reviewer for this helpful suggestion and agree that protein-level validation could provide additional support.

However, the primary aim of this experiment was to establish a system with reduced A-to-I editing in order to assess the performance of our analysis pipeline. In this context, ADAR knockdown serves as a means to modulate editing levels rather than an endpoint in itself.

To validate this, we combined multiple complementary approaches, including RT-qPCR and RNA-seq to confirm reduced Adar/Adarb1 expression, EndoV assays to directly assess inosine-containing RNAs, and importantly, orthogonal validation of editing levels using Nanopore direct RNA sequencing. These consistently demonstrate a clear reduction in editing upon knockdown.

While we agree that Western blot analysis would be informative, we believe that the combination of expression-level and multiple independent functional readouts of editing sufficiently addresses the underlying concern.

Response to Reviewers' Comments

<u>Point 2.</u> The reviewer asks: *"Figure 2 should include a panel showing the relevant dsRNA structures marked with the corresponding editing hotspots. Is there anything special about the editing hotspots?"*

We thank the reviewer for this insightful suggestion.

In the revised manuscript, we have opted not to include a direct mapping of editing hotspots onto a single dsRNA structure. This is because our approach is inherently population-based, aggregating signals across many B2 RNA molecules that differ in sequence (e.g., insertions, deletions, and mismatches). As a result, there is no single universal secondary structure that can accurately represent all contributing transcripts.

While we use the consensus sequence for positional reference, structural predictions based on this sequence do not fully capture the variability present in the underlying RNA population, which limits the interpretability of direct structure–editing overlays.

In addition, it remains unclear whether the observed editing arises from intramolecular dsRNA structures within individual B2 RNAs or from intermolecular pairing (e.g., inverted repeat interactions), as previously reported. This further complicates direct structural interpretation and is now discussed in the revised manuscript.

We now emphasize in the text that, while some editing hotspots fall within regions previously associated with dsRNA formation, a precise structural assignment would require dedicated future analysis.

The newly added text is now: "This new finding raises the potential that intramolecular double strand RNA formations within the SINE B2 RNA, that have been previously described in case of the B2 consensus sequence (Espinoza et al., 2007; Podbevsek et al., 2018; Sharma et al., 2024), may also play a role in the editing process beyond the mechanism of inverted SINE transcripts. However, a precise structural assignment would require dedicated future structural analysis of multiple different B2 RNAs, because structural predictions based only on the consensus sequence do not fully capture the variability present in the underlying RNA population, which limits the interpretability of direct structure–editing overlays."

<u>Point 3.</u> The reviewer asks: *"Instead of combined knockdown ("AntiADAR"), it would be useful to examine ADAR1 and ADAR2 individually. The authors wrote that "both enzymes demonstrably can edit B2 RNAs, though with potentially different efficiencies". Which sites are ADAR1 targets and which are ADAR2 targets? Moreover, Alu repeats in human are predominantly ADAR1 targets, so it's interesting that ADAR2 appears to play a bigger role in the mouse."*

We thank the reviewer for this valuable comment. In this study, our primary aim was to establish a system with reduced A-to-I editing in order to evaluate the performance of our analysis framework. For this reason, we employed combined Adar/Adarb1 knockdown to ensure a robust reduction in editing activity and to avoid potential compensatory effects between the two enzymes.

Accordingly, the focus of the manuscript is on editing outcomes rather than enzyme-specific contributions. While distinguishing ADAR1- versus ADAR2-specific editing sites is an important and interesting question, addressing this would require a dedicated experimental design and is beyond the scope of the current study.

We note that previous work, including publicly available datasets (e.g., ADAR mutant models such as those reported in Genome Research https://genome.cshlp.org/content/30/8/1107.full#ref-43), has begun to explore enzyme-specific targeting in repetitive elements. We have now clarified this point in the Discussion.

The newly added text is now: "While both ADAR1 and ADAR2 are known to contribute to A-to-I editing, the present study does not distinguish enzyme-specific targeting. Our use of combined Adar/Adarb1 knockdown was designed to ensure a robust reduction in overall editing and avoid potential compensatory effects between the two enzymes. Dissecting ADAR1- versus ADAR2-specific contributions to B2 RNA editing remains an important direction for future work."

<u>Point 4.</u> The reviewer asks: *"Instead of xPORE, the authors should use deep learning models that can detect A-to-I editing in native RNA directly?"*

We thank the reviewer for this valuable suggestion.

We agree that emerging deep learning–based approaches for detecting A-to-I editing from Nanopore data represent an important advancement, particularly in the context of the new Nanopore RNA chemistry and respective base caller released recently that can identify inosines. However, this base caller is not readily applicable to the R9 chemistry used in this study, for which current publicly available analysis approaches for Inosine remain limited to non-ANN based approaches such as xPORE. In this context, our use of Nanopore data was intended as an orthogonal validation of editing signals, complementing the Illumina-based analysis rather than serving as a standalone detection method. The observed concordance between platforms supports the robustness of our findings.

Given these technical considerations, we believe that developing deep learning model for base calling of inosine to our dataset would not provide additional insight at this stage, although this represents an important direction for future studies working with the new RNA chemistry flowcells.

<u>Point 5.</u> The reviewer remarks: "*Figure 3B: It seems like A-to-I editing starts off being higher in APP mice and then decreases over lifespan and drops to a level lower than that in wildtype controls by 12 months. Are there any clues in the gene expression data that might provide an explanation to this pattern?"*

We thank the reviewer for this insightful question.

Response to Reviewers' Comments

In the revised manuscript, we have addressed this point by including a new Supplementary Figure 9, which presents Adar and Adarb1 expression levels at 6 and 12 months. These time points were specifically selected because they flank the observed inversion in editing levels, where editing shifts from being higher in APP mice (6 months) to lower in APP mice (12 months) relative to controls.

The data show induced expression of both enzymes at 6 months APP vs the WT compared to 12 months, consistent with the observed differences in A-to-I editing. This suggests that the late-stage reduction in editing may be, at least in part, attributable to changes in ADAR expression. This point is now discussed in the revised manuscript.

The newly added text is now: "For example, changes in ADAR expression are likely contributing to the observed increase in B2 RNA editing in APP mice relative to wild type at 6 months, a difference that is no longer observed at 12 months. As shown in Supplementary Figure 9, Adar and Adarb1 expression levels are elevated in APP mice compared to wild type at 6 months, whereas this difference between genotypes is no longer present at 12 months. This pattern mirrors the editing data, where higher A-to-I editing in APP mice relative to wild type at 6 months is lost at 12 months. Together, these observations suggest that changes in ADAR expression may contribute, at least in part, to the stage-specific differences in B2 RNA editing between APP and wild type animals, although as mentioned above more targeted studies are required to dissect any distinct roles of Adar and Adarb1 in this."

Minor comment:

<u>Point 6.</u> The reviewer asks: *" Figure 2E should appear earlier. Editing analysis should follow only after confirmation of successful knockdown."*

The reviewer's suggestion has been now fully implemented.

REVIEWER 2

Reviewer #2 (Comments to the Authors (Required)):

1.Summary

This manuscript by Mitchell, Saville, and colleagues presents two interconnected contributions: a novel repeat-aware bioinformatics framework for quantifying position-specific adenosine-to-inosine (A-to-I) RNA editing within SINE B2 non-coding RNAs, and the application of this framework to reveal a previously unrecognized epitranscriptomic response to amyloid beta neurotoxicity.

Response to Reviewers' Comments

The methodological advance centers on a "REPome"-based approach that circumvents the well-known problems of multi-mapping reads and sequence heterogeneity that plague standard genome-aligned editing pipelines when applied to repetitive elements. By collapsing identical B2 sequences into unique representatives and treating the expressed population as a collective, the authors achieve population-level, position-specific editing quantification that standard methods cannot reliably deliver. Applying this framework, the authors identify discrete A-to-I editing hotspots within B2 mm1a RNAs - concentrated in regions predicted to form double-stranded RNA structures - whose editing levels are selectively elevated during the earliest stages of amyloid beta pathology. This finding is reproduced across two independent mouse models (APPNL-G-F and APPB6.APP/PS1), in hippocampal cell culture exposed to acute amyloid beta peptide toxicity, and validated using an orthogonal sequencing technology (Oxford Nanopore direct RNA-seq). Functional perturbation via siRNA-mediated knockdown of Adar/Adarb1 confirms ADAR enzyme dependence of the identified editing events and demonstrates that reducing ADAR activity suppresses expression of B2 RNA-regulated stress-response genes (B2 SRGs). Together, the work defines a regulatory axis linking amyloid beta toxicity → ADAR upregulation → site-specific B2 RNA editing → B2 SRG expression, with potential relevance to conserved mechanisms in Alzheimer's disease.

This reviewer generally remarks that "*This is a significantly improved manuscript relative to its initial submission. The authors have responded substantively to prior reviewer concerns by replacing the non-specific 8-AzaA inhibitor with siRNA knockdown experiments, adding EndoV biochemical validation, incorporating Nanopore direct RNA-seq as an orthogonal sequencing approach, including an independent external mouse dataset, and refining the bioinformatics pipeline to improve positional resolution at the subfamily level. These additions substantially strengthen both the methodological and biological claims.*"

We appreciate the encouraging comments made by this reviewer.

***2. Assessment of Main Points ***

<u>**Point 1.**</u> **The reviewer generally remarks that:** "*The REPome-based pipeline outperforms standard genome-aligned approaches for B2 RNA editing quantification. Assessment: Well supported. The comparison presented in Supplementary Figure 2 is informative and directly addresses the prior reviewer concern. The authors demonstrate that genome-based alignment captures substantially fewer reads mapping to B2 elements (Suppl. Fig. 2A), and that the conventional edit-then-aggregate approach produces an artifactually flat, positionally uninformative editing distribution that fails to detect the expected ADAR-dependent signal (Suppl. Fig. 2C). By contrast, the REPome-based, group-then-edit-rate approach faithfully recovers the reduction in editing caused by Adar/Adarb1 knockdown, with clear positional hotspots consistent with known dsRNA-forming regions of B2 RNA (Suppl. Fig. 2B, Fig. 2B-D). The logic underpinning the population-level proportion approach is sound: by treating variability among B2 copies as a distributional property rather than a mapping problem, the method avoids both read loss and locus misassignment. No additional experiments are required to support this claim.*"

Response to Reviewers' Comments

The reviewer then proposes: *" One clarification that would benefit readers: the Methods section describes filtering for mm1a elements specifically after initially constructing the REPome from mm1a, mm1t, and mm2 sequences. It would be helpful to explicitly state in the Results or Methods whether reads that align better to mm1t or mm2 elements are excluded prior to the position-specific analysis, and how cross-subfamily alignment ambiguity is handled. This does not require new experiments - a sentence or two of clarification suffices."*

We thank the reviewer for this helpful clarification request and for the positive assessment of our approach.

In the revised manuscript, we clarify that, following alignment to the REPome constructed from mm1a, mm1t, and mm2 sequences, the position-specific analysis shown in the main figures is restricted to reads mapping to the mm1a subfamily, with reads aligning to mm1t and mm2 excluded at this stage to ensure consistent positional interpretation.

At the same time, we note that all three subfamilies (mm1a, mm1t, and mm2) are included in the global editing rate comparisons presented in Supplementary Figure 8, allowing assessment of broader subfamily-level trends. This distinction is now clarified in the manuscript.

We have also clarified in the revised Methods that reads are not excluded based on subfamily identity; rather, they are segmented by B2 subfamily (mm1a, mm1t, mm2) and edit rates are computed independently for each group. The position-specific analyses shown in the main figures are based on the mm1a-derived edit rates, while other subfamilies are retained for comparative analyses (e.g., Supplementary Figure 8). We further clarified that our alignment strategy (bwa aln to the REPome) yields a single read-to-reference assignment, avoiding cross-subfamily ambiguity. This has now been explicitly described in the Methods section as well as in the Results.

The newly added text is now: "In this context, it should be noted that position-specific editing analyses presented in the main figures are restricted to reads mapping to the mm1a subfamily, which most closely matches the canonical B2 consensus and allows consistent positional interpretation."

The newly added text in METHODS is now: "Additionally, we calculated this rate separately for each B2 subfamily, selected B2 Mm1a elements, and then we examined only positions within the B2 Mm1a consensus sequence that contain an A. In particular, REDItools tables generate base composition summaries for each unique REPome reference sequence, preserving B2 subfamily identity. For each position in each sample, observed and expected base proportions are calculated for mm1a, mm1t, and mm2 elements, and edit rates are computed independently for each subfamily. Analyses presented in the main figures focus on the mm1a-derived edit rates, using positions corresponding to adenosines in the mm1a consensus sequence. Importantly, alignment using bwa aln produces a single read-to-reference assignment, thereby avoiding cross-subfamily alignment ambiguity."

Response to Reviewers' Comments

<u>Point 2.</u> The reviewer generally notes that "*A-to-I editing is increased at specific B2 RNA positions during early amyloid beta pathology in the mouse hippocampus Assessment: Well supported, with minor caveats regarding statistical power at the 3-month timepoint.*
The cumulative and position-specific editing analyses in APP versus WT mice at 3, 6, and 12 months (Fig. 3B-D) present a coherent picture: editing is elevated early (3m and 6m) and this increase reverses at the terminal stage (12m), consistent with a dynamic epitranscriptomic response rather than a static feature. The eight positions identified as significantly differentially edited (Suppl. Fig. 3) are reproducible across datasets, which is an important strength." **The reviewer then suggests:** *"One limitation worth acknowledging more explicitly in the text is that the 3-month WT group contains only n=2 animals, which limits statistical power for that comparison. The authors note this in the figure legend but the Discussion does not explicitly caveat the 3-month findings accordingly. Given that the pre-symptomatic timepoint is arguably the most interesting from a biomarker standpoint, a brief acknowledgment of this limitation in the Discussion would be appropriate. This is a text change only."*

We thank the reviewer for this important point.

We agree that the limited sample size in the 3-month WT group (n=2) reduces statistical power at this early time point. While the observed trend is consistent with later stages and across independent datasets, we have now explicitly acknowledged this limitation in the Discussion and interpret the 3-month findings with appropriate caution.

The newly added text is now: *"*While increased editing is observed at early stages of pathology, it should be noted that the 3-month WT group includes a limited number of samples (n=2), which reduces statistical power for this comparison. Therefore, although the observed trend is consistent with findings at later time points and across independent datasets, the results at this pre-symptomatic stage should be interpreted with appropriate caution.*"*

<u>Point 3.</u> The reviewer remarks that "*Findings replicate in an independent external dataset (APPB6.APP/PS1 model) Assessment: Well supported. The replication of the elevated editing pattern in whole-brain tissue from an independent mouse model generated by a different laboratory (GEO: GSE136861), presented in Supplementary Figures 4 and 5, is a meaningful addition. The overlap of significantly edited positions between the two models (Suppl. Fig. 4D) and the parallel direction of editing changes at the eight-position subset (Suppl. Fig. 5) substantially mitigate concerns about model-specificity or laboratory-specific batch effects. No additional experiments are required."* **The reviewer then suggests: "***It would be worth noting explicitly that this external dataset uses whole-brain tissue rather than hippocampus-specific dissection, and that the transgene approach (human APP and PSEN1 overexpression) differs genetically from the APP knock-in strategy. The authors are aware of these differences but they are only briefly mentioned. Acknowledging these distinctions more directly in the Results - and noting them as a strength of cross-model replication rather than a confound - would help readers contextualize the generalizability of the findings."*

We thank the reviewer for this thoughtful comment.

In the revised manuscript, we now explicitly highlight that the external dataset (GSE136861) differs from our primary dataset in both tissue source (whole brain vs. hippocampus) and genetic model (APP/PSEN1 transgenic overexpression vs. APP knock-in). Importantly, we emphasize that the replication of editing patterns across these distinct experimental contexts strengthens the generalizability of our findings, rather than representing a confounding factor. These clarifications have now been incorporated into the Results section.

The newly added text is now: "Importantly, this external dataset differs from our primary cohort in both tissue source and genetic background, comprising whole-brain RNA-seq data rather than hippocampus-specific samples and employing a transgenic APP/PSEN1 overexpression model as opposed to the APP knock-in strategy used here. The observation of consistent editing patterns across these distinct experimental settings supports the robustness of the identified A-to-I editing changes."

Point 4. The reviewer remarks that "*Nanopore direct RNA-seq independently supports elevated adenosine modification at B2 RNA positions Assessment: Supportive, with an important and appropriately acknowledged caveat regarding modification specificity. The Nanopore data (Fig. 4) provide a valuable orthogonal line of evidence. Both the Xpore-computed modification rate distributions and the raw Nanopolish signal density plots at the eight identified positions show directionally consistent patterns: higher modification signals in Scramble versus AntiAdar cells, and in 6-month APP versus WT hippocampi. The KS statistics (KS=0.001 and KS=0.002, respectively) indicate meaningful distributional differences. The authors appropriately note in the Discussion that the R9.4.1 flowcell chemistry cannot unambiguously distinguish inosine from other RNA modifications, and that other base modifications or ADAR-independent crosstalk cannot be excluded. This caveat is honest and scientifically appropriate". **The reviewer then suggests: "***Position 112 showing no difference in Nanopore signal despite being identified as significantly edited by Illumina sequencing is also acknowledged - the authors should briefly speculate in the Discussion whether this reflects lower coverage at that position, a local sequence context unfavorable for nanopore signal discrimination, or a potential false positive in the Illumina-based analysis. No additional experiments are needed; this is a minor interpretive addition."*

We thank the reviewer for this thoughtful comment and for recognizing the value of the orthogonal Nanopore-based validation.

We would first like to note that there was a small error in the previous version of the manuscript. As it was correctly shown in Figure 4C and mentioned in its legend , the position that did not show a clear difference in Nanopore signal distributions despite being identified by the Illumina-based analysis is position 70 and not position 112 as erroneously was mentioned in the text. This has now been corrected.

In the revised manuscript, we now explicitly discuss the potential reasons why position 70, despite being identified as significantly edited in the Illumina-based analysis, did not show a clear difference in Nanopore signal distributions. In particular, we note that Nanopore-based detection

sensitivity can vary substantially depending on local sequence context and k-mer composition, which may limit the ability of the R9.4.1 chemistry and associated signal models to resolve modifications at specific positions. In addition, the specific Nanopore chemistry and analysis framework used in this study cannot unambiguously distinguish inosine from other RNA modifications, raising the possibility that overlapping or interacting RNA modification signals may partially obscure position-specific discrimination. Local coverage differences and weaker signal perturbation at this site may have further contributed to the lack of detectable separation.

The newly added text is now: "which could also partially explain the discrepancy between the illumina and nanopore results at position 70……. Moreover, the fact that position 70 identified as significantly edited by the Illumina-based analysis did not show a corresponding separation in Nanopore signal distributions, may reflect limitations of the R9.4.1 Nanopore chemistry and associated signal models, as modification detectability depends on local sequence and k-mer context, while local read coverage or signal perturbation at this site may also have limited discrimination power."

Point 5. **The reviewer remarks that** "*ADAR knockdown validates pipeline sensitivity and links editing to B2 SRG expression. Assessment: Well supported. The siRNA knockdown of Adar and Adarb1 (Fig. 2) is a significant improvement over the previously used 8-AzaA inhibitor. Knockdown efficiency is confirmed at both the transcript level (Fig. 2E, RT-qPCR; Suppl. Fig. 1, RNA-seq) and functionally via the EndoV inosine-cleavage assay (Fig. 2F). The EndoV result is particularly valuable as it provides a sequence-independent biochemical confirmation that inosine-containing B2 RNA transcripts are depleted following ADAR knockdown. The correlation between Adar/Adarb1 expression and cumulative editing rate across individual replicates (Suppl. Fig. 1, R=0.78 and R=0.91) is compelling. The connection between ADAR activity and B2 SRG expression (Fig. 6C) is logically presented and the directionality is consistent: knockdown reduces editing and correspondingly reduces B2 SRG expression. The per-replicate correlations between cumulative editing and B2 SRG expression (Suppl. Fig. 7) further support this coupling. The authors are appropriately cautious about mechanistic interpretation, correctly noting that the exact causal pathway linking editing to SRG expression will be addressed in a forthcoming manuscript. No additional experiments are required for the claims made in this paper..*"

We thank the reviewer for these encouraging comments and for recognizing the value of the combined molecular, biochemical, and orthogonal sequencing validation approaches used in this study.

Point 6. **The reviewer remarks that** "*Acute amyloid beta peptide exposure recapitulates the in vivo editing pattern in HT-22 cells Assessment: Well supported. The in vitro model (Fig. 5) shows that 6-hour exposure to amyloid beta 1-42 peptides increases B2 RNA editing at the same positional hotspots identified in APP mice, with directionally consistent changes at the eight-position subset (Suppl. Fig. 6). The consistency across in vivo and in vitro systems - and across the acute toxicity model and the chronic pathology model - is a meaningful strength of the dataset as a whole..*"

Response to Reviewers' Comments

We thank the reviewer for this encouraging assessment and for recognizing the consistency of the identified editing patterns across both the in vivo and in vitro amyloid beta toxicity models.

***3. Additional Issues ***

Text and presentation:
• The abstract refers to "hippocampal or neocortical tissue from two independent mouse models." The neocortex mention appears to refer to the whole-brain external dataset. The authors should clarify in the abstract and Results that the external validation dataset uses whole-brain tissue (which includes but is not restricted to neocortex), to avoid overstating regional specificity.

We thank the reviewer for pointing out this ambiguity.

In the revised manuscript, we have clarified the wording in the Abstract and Results section to more accurately describe the external validation dataset as whole-brain tissue rather than neocortical tissue specifically.

The revised text now reads: "Elevated B2 RNA editing is consistently observed across hippocampal tissue from one mouse model of amyloid beta accumulation, whole-brain tissue from an independent similar mouse model…"

• The Discussion (page 13) states this is "the first study in hippocampus to identify specific positions within SINE RNAs that act as hotspots of A-to-I editing." Given that the external validation dataset uses whole-brain tissue and the in vitro work uses a hippocampal-derived cell line, the phrasing should perhaps be broadened slightly, or more carefully qualified to distinguish hippocampal tissue findings from cell line findings.

We thank the reviewer for this helpful suggestion.

In the revised manuscript, we have slightly broadened and clarified this statement to distinguish between the hippocampal tissue findings, the whole-brain validation dataset, and the hippocampal-derived cell line experiments.

The revised text now reads: "This is the first study to identify specific positions within SINE RNAs that act as hotspots of A-to-I editing in hippocampus and related amyloid beta toxicity models"

• Figure 4C legend refers to "position 70" as showing identical distributions corresponding to non-detectable modification changes. However, position 70 does not appear in the list of the eight significantly edited positions in Figure 3D, which is consistent. It would be helpful to add a brief parenthetical noting that position 70 serves as an internal negative control in this panel, to assist readers in interpreting the layout.

Response to Reviewers' Comments

We apologize for the confusion. There was a discrepancy between this legend and what was initially mentioned in the text (position 112 instead of position 70, please see response to point 4 above). Position 70 is differentially edited based on the Illumina sequencing data (Fig 3D and Suppl Fig 3B). As noted in point 4, the correction in the text has now been made.

• *The Methods section describes REDItools parameters including setting minimum editing frequency to 0 for "maximum sensitivity." While the rationale for other parameters is explained, the authors might briefly note that setting -n 0 could in principle introduce false positives at positions with very low coverage, and explain how the subsequent population-based approach and the editing rate threshold of* $>10^{-4}$ *mitigate this concern.*

We thank the reviewer for this important comment.

In the revised manuscript, we now further clarify the rationale underlying the permissive REDItools parameter selection. In our framework, REDItools is not used as a conventional editing detection tool, but rather exclusively as an aggregator of aligned read and reference base composition information. Consequently, the downstream estimation of editing rates is not based on direct REDItools edit calls, but instead on independently calculated population-level observed and expected base proportions across the REPome.

We further clarify that, although permissive REDItools thresholds could in principle increase false-positive editing detection in low-coverage regions, this issue is mitigated in our approach because edit rates are computed independently at the level of read and reference populations. Regions with higher read coverage naturally contribute proportionally more strongly to the observed base distributions, while reference ratios are calculated only from regions with coverage, thereby reducing the influence of sparse or unsupported positions. These clarifications have now been added to the Methods section of the revised manuscript.

The revised Methods text now reads: "REDItools parameters were selected to maximize recovery of aligned read information from repetitive B2 RNA regions. In this framework, REDItools was used exclusively to aggregate read and reference base composition data rather than to directly perform RNA editing calling. Consequently, the permissive parameter settings were applied to maximize sensitivity at the level of underlying alignment information. Although such settings could theoretically increase false-positive edit detection in low-coverage regions in conventional REDItools-based analyses, this issue is mitigated in our population-based framework because editing rates are computed independently from aggregated observed and expected base distributions across the REPome. Read-derived base proportions are calculated across all aligned reads, naturally weighting positions according to sequencing coverage, whereas reference-derived proportions are computed only from reference regions with detectable coverage. This approach minimizes the contribution of unsupported low-coverage positions while preserving sensitivity across repetitive SINE-derived transcripts."

Statistics:

• *Supplementary Figures 3B, 5B, and 6B present one-tailed Student's t-tests. The choice of one-*

tailed testing is presumably justified by the directional hypothesis (editing is increased in disease/amyloid conditions). This should be stated explicitly in the figure legends or a consolidated statistical methods section, as one-tailed tests are sometimes viewed critically without clear a priori justification.
• The authors report KS test values for cumulative distribution comparisons throughout but do not specify whether these are two-sample KS tests. This should be clarified in the Methods.

We thank the reviewer for this important comment.

In the revised manuscript, we clarified the statistical framework used throughout the study. Specifically, we now state that these tests were based on directional hypotheses derived from the experimental design. In the siRNA-mediated Adar/Adarb1 knockdown experiments, the directional hypothesis assumed reduced editing following ADAR depletion and therefore higher editing levels in the Scramble controls.

We additionally clarified that cumulative distribution comparisons were performed using two-sample Kolmogorov–Smirnov (KS) tests, which evaluate nondirectional differences between distributions. These clarifications have now been incorporated into the Methods section of the revised manuscript as follows: "Student's t-tests (where applicable) were based on directional hypotheses derived from the experimental design. In the siRNA-mediated Adar/Adarb1 knockdown experiments, the directional hypothesis assumed reduced editing following ADAR depletion and therefore higher editing levels in the Scramble controls. Cumulative distribution comparisons were performed using two-sample Kolmogorov–Smirnov (KS) tests, which evaluate nondirectional differences between distributions."

Minor:
• Page 7, Results: "Adar1b1" appears to be a typographical error for "Adarb1."

This has now been corrected.

• The GEO accession number for the new Illumina and Nanopore data (GSE316378) is provided in the Methods, which is appreciated. Authors should confirm this accession is publicly accessible at the time of publication.

GEO accession numbers have been updated and will be publicly available.

• Supplementary Figure 8 comparing editing across mm1a, mm1t, and mm2 subfamilies is informative and the observation that mm2 displays divergent editing behavior is interesting. The authors might consider adding one sentence in the Discussion noting whether the absence of elevated editing in mm2 elements - which lack the conserved ribozyme motifs - could be consistent

with a model linking editing sites to functional RNA secondary structures. This is a minor interpretive point that does not require new data.

We thank the reviewer for this insightful observation.

We agree that the divergent editing pattern observed in mm2 elements relative to mm1a/mm1t is biologically interesting and may reflect the distinct sequence architecture of mm2 elements, including the absence of conserved motifs present in the canonical B2 consensus.

In the revised manuscript, we now mention this point more explicitly in the Discussion section, where we note that differences in sequence organization between B2 subfamilies may contribute to distinct editing behaviors and potentially distinct functional properties related to RNA secondary structure formation and regulation. We further emphasize that these observations support the need for future subfamily-specific functional studies of SINE RNA editing.

The revised text now reads:"These sequence differences include the absence of several conserved motifs present in the canonical B2 consensus, which may contribute to altered RNA secondary structure formation and consequently distinct editing behaviors."

Reviewer #2 (Final Recommendation):

I am pleased to endorse this manuscript for publication in its current state. The authors have produced a rigorous, well-validated study that makes a genuine methodological contribution to the field of RNA editing analysis and uncovers a biologically compelling epitranscriptomic response to amyloid beta neurotoxicity. The revisions undertaken in response to prior review have been thorough and have meaningfully strengthened the manuscript. The points raised above are minor in nature and can be addressed at the discretion of the authors and editors without further review. I commend the authors for the quality and scope of this work.

June 1, 2026

RE: Life Science Alliance Manuscript #LSA-2026-03668-TR

Dr. Athanasios Zovoilis
University of Manitoba
Biochemistry and Medical Genetics
Canada

Dear Dr. Zovoilis,

Thank you for submitting your Research Article entitled "Differential A to I editing of SINE B2 RNAs unveils an epitranscriptome response to Aβ neurotoxicity". It is a pleasure to let you know that your manuscript is now accepted for publication in Life Science Alliance. Congratulations on this interesting work.

The final published version of your manuscript will be deposited by us to PubMed Central upon publication.

Your manuscript will now progress through copyediting and proofing. It is journal policy that authors provide original data upon request.

Your article will publish open access upon publication under a CC-BY license.

Reviews, decision letters, and point-by-point responses associated with peer-review at Life Science Alliance will be published, alongside the manuscript. If you do want to opt out of having the reviewer reports and your point-by-point responses displayed, please let us know immediately.

IMPORTANT: If you will be unreachable at any time, please provide us with the email address of an alternate author. Failure to respond to routine queries may lead to unavoidable delays in publication.

Scheduling details will be available from our production department. You will receive proofs shortly before the publication date. Only essential corrections can be made at the proof stage so if there are any minor final changes you wish to make to the manuscript, please let the journal office know now.

DISTRIBUTION OF MATERIALS:
Authors are required to distribute freely any materials used in experiments published in Life Science Alliance. Authors are encouraged to deposit materials used in their studies to the appropriate repositories for distribution to researchers.

You can contact the journal office with any questions, contact@life-science-alliance.org

Again, congratulations on a very nice paper. I hope you found the review process to be constructive and are pleased with how the manuscript was handled editorially. We look forward to future exciting submissions from your lab.

Sincerely,

Tim Fessenden
Scientific Editor
Life Science Alliance
